# Polyphenolic Profile of *Callistemon viminalis* Aerial Parts: Antioxidant, Anticancer and In Silico 5-LOX Inhibitory Evaluations

**DOI:** 10.3390/molecules26092481

**Published:** 2021-04-24

**Authors:** Shahenda Mahgoub, Nashwa Hashad, Sahar Ali, Reham Ibrahim, Ahmed M. Said, Fatma A. Moharram, Mohamed Mady

**Affiliations:** 1 Biochemistry and Molecular Biology Department, Faculty of Pharmacy, Helwan University, Ein-Helwan, Helwan, Cairo 11795, Egypt; ganah_nour@yahoo.com; 2 Department of Pharmacognosy, Faculty of Pharmacy, Helwan University, Ein Helwan, Cairo 11795, Egypt; alsayednashwa@gmail.com (N.H.); reham_bassyoni@pharm.helwan.edu.eg (R.I.); mohamedsaid_1985@pharm.helwan.edu.eg (M.M.); 3 Department of Pharmaceutical Organic Chemistry, Faculty of Pharmacy, Helwan University, Ein-Helwan, Helwan, Cairo 11795, Egypt; 4 Department of Chemistry, University at Buffalo, The State University of New York, Buffalo, NY 14260, USA

**Keywords:** antioxidant, cytotoxicity, C. viminalis, flavonoids, polyphenolic, 5-LOX, molecular modelling

## Abstract

Five new compounds *viz* kaempferol 3-*O*-(4″-galloyl)-*β*-d-glucopyranosyl-(1‴→6″)-*O*-β-d-glucopyranoside (**1**), kaempferol 3-*O*-*β*-d-mannuronopyranoside (**2**), kaempferol 3-*O*-*β*-d-mannopyranoside (**3**), quercetin 3-*O*-*β*-d-mannuronopyranoside (**4**), 2, 3 (S)- hexahydroxydiphenoyl]-d-glucose (**5**) along with fifteen known compounds were isolated from 80% aqueous methanol extract (AME) of *C. viminalis*. AME and compounds exerted similar or better antioxidant activity to ascorbic acid using DPPH, O_2_^−^, and NO inhibition methods. In addition, compounds **16**, **4**, and **7** showed cytotoxic activity against MCF-7 cell lines while **3**, **7** and **16** exhibited strong activity against HepG2. An in silico analysis using molecular docking for polyphenolic compounds **2**, **3**, **7**, **16** and **17** against human stable 5-LOX was performed and compared to that of ascorbic acid and quercetin. The binding mode as well as the enzyme-inhibitor interactions were evaluated. All compounds occupied the 5-LOX active site and showed binding affinity greater than ascorbic acid or quercetin. The data herein suggest that AME, a source of polyphenols, could be used against oxidative-stress-related disorders.

## 1. Introduction

Genus *Callistemon* (commonly known as bottlebrushes) belongs to the family Myrtaceae and comprises about 34 species, it is widely distributed across the world [1]. *Callistemon viminalis* (weeping bottlebrush) is found as a small tree or shrub distributed around the world particularly in tropical Asia, Australia, Sri Lanka, South America, and India [2] as well as cultivated in Egypt. It was planted as an ornamental plant, and as a source of essential oil [3]. *C. viminalis* is traditionally used for treating skin infections, hemorrhoids, gastroenteritis, diarrhea, and respiratory conditions [1,4]. Among the plant components, secondary metabolites like, phenolic compounds are of exceptional interest due to their noticeable health-related properties. From the chemical point of view, there are few reports about the phenolic compounds of *C*. *viminalis* which concentrated on the presence of flavonoids [5,6], tannins [6], and phenolic acid [3,6,7]. In addition, several phloroglucinol compounds were identified [8,9,10]. Moreover, some studies have demonstrated that the alcoholic extracts of the leaves, aerial parts, flower, bark, and fruits of *C*. *viminalis* possessed different biological activities as cytotoxic [5,11,12], antioxidant [6,7,11,12,13,14,15], hepatoprotective [6], antidiabetic [16], and analgesic activities [17]. Reactive oxygen species (ROS) are generated by different physiological processes and they have important functions in organisms; one of them is the defense function, as shown in the activation polymorphonuclear neutrophils by exogenous or endogenous motivations, which is followed by increasing the oxygen consumption and ROS production cascade leading to generation of other free radicals as superoxide anion (O_2_^−^) and hydrogen peroxide (H_2_O_2_) [18]. ROS generation reactions can be elevated under certain conditions resulting in oxidative stress and tissues damage [19].

Though the NADPH oxidase family is well known as a main source of non-mitochondrial ROS in several cells, arachidonic acid (AA) metabolism by lipoxygenases (LOX) also plays a part in the generation of ROS in a variety of cells [20]. Lipoxygenases, nonheme iron-containing enzymes, catalyze the peroxidation/oxidation of the lipids (polysaturated fatty acids) to form various reactive inflammatory mediators. These enzymes were found to significantly contribute to the pathogenesis of various diseases including cancers [21]. One important enzyme from the lipoxygenase family is 5-lipoxygenase (5-LOX) was found to trigger the inflammatory response through the biosynthesis of leukotrienes (LTs) [22]. During this process ROS are also formed as by-products [23]. Recent studies revealed that the overexpression of 5-LOX significantly contributes to certain autoimmune diseases, i.e., rheumatoid arthritis as well as severe allergic reactions, i.e., asthma [24]. In addition, several clinical and preclinical studies found out that the overexpression of 5-LOX was found to also significantly result in carcinogenesis [25]. Recent studies revealed that 5-LOX plays an important role in tumor formation through stimulating cell proliferation [26], apoptosis inhibition [27], excessive production of various mutagens [28]. 5-LOX was found to have a profound effect on the prognosis of several types of cancer, i.e., breast [25,29], colon, liver [30], and pancreatic cancer [31]. The inhibition of 5-LOX could be a potential therapeutic pathway to treat and prevent cancer formation via blocking the oxidative and inflammatory pathways [32].

Antioxidants from natural sources have been receiving great attention from consumers and researchers due to their several pharmacological activities as well as their ability to protect the organism from oxidative stresses caused by ROS [33]. Polyphenolic compounds are gaining significance due to their contribution to human health along with their several biological activities as antioxidant, antimutagenic, anticarcinogenic, and cytoprotective activities [34]. Considering ROS participation in several pathologic processes and the different use of natural polyphenols as therapeutics, this study was conducted to assess the polyphenolic profile of 80% aqueous methanol extract (AME) of *C. viminalis* aerial parts for ROS scavenging agents and evaluate if through their antioxidant properties, the extract and the pure isolated compounds can protect cells against oxidative stress. In addition, anticancer activity of AME along with isolated compounds against MCF-7 and HepG2 cancer cell lines were investigated. In addition, using molecular docking an in silico analysis for the binding mode and affinity of six polyphenolic compounds was performed and was compared to that of ascorbic acid and quercetin as reference drugs.

## 2. Results and Discussion

### 2.1. Chemical Structure Elucidation of Compounds Isolated from C. viminalis Aerial Parts

**Figure 1:**
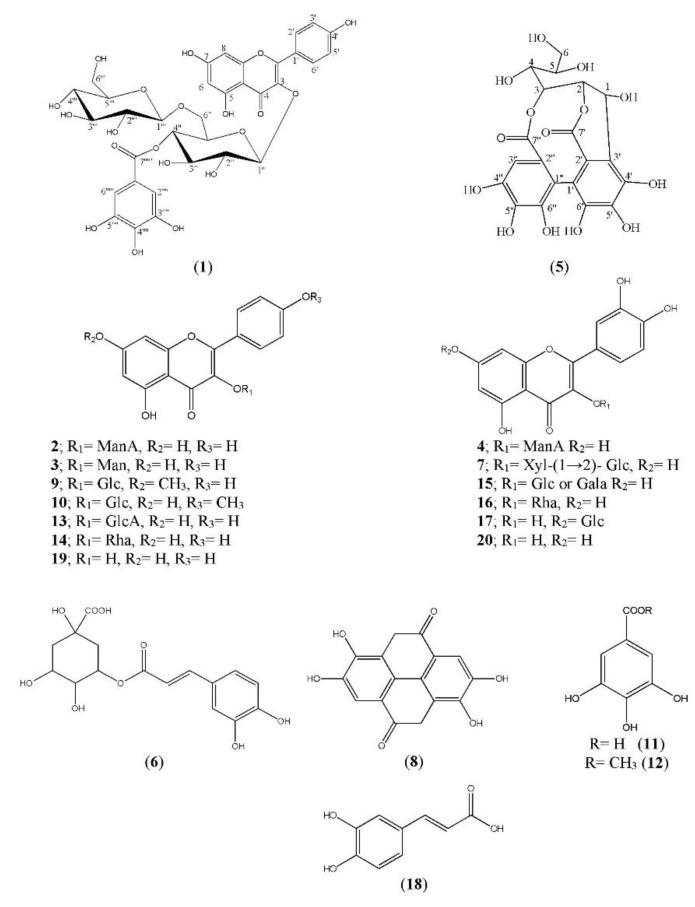
Structures of polyphenolic compounds from *C. viminalis* aerial parts. Glc: Glucose, Gala: Galactose, GlcA: Glucuronic acid, Man: Mannose, ManA: Mannuronic acid, Rha: Rhamnose.

Fractionation of the AME of *C. viminalis* aerial parts on polyamide column followed by purification of the compounds on successive cellulose and Sephadex LH-20 columns resulted in isolation of five new compounds *viz* kaempferol 3-*O*-(4″-galloyl)-*β*-d-glucopyranosyl-(1‴→6″)-*O*-*β*-d-glucopyranoside (**1**), kaempferol 3-*O*-*β*-d-mannuronopyranoside (**2**); kaempferol 3-*O*-*β*-d-mannopyranoside (**3**), quercetin 3-*O*-*β*-d-mannuronpyranoside (**4**) 2, 3 (*S*)-hexahydroxydiphenoyl]-d-glucose (**5**), together with four known kaempferol glycoside: kaempferol 7-methyl ether 3-*O*-*β*-d-glucopyranoside (**9**), kaempferol 4′ methyl ether 3-*O*-*β*-d-glucopyranoside (**10**), kaempferol 3-*O*-*β*-d-glucuronopyranoside (**13**) kaempferol 3-*O*-*β*-d-rhamnopyranoside (**14**) [35,36,37], four quercetin glycoside *viz* quercetin 3-*O*-*β*-d-xylopyranosyl-(1‴→2″)-*O*-*β*-d-glucopyranoside (**7**) [38,39], isoquercetin (**15**), quercitrin (**16**), quercetin 7-*O*-*β*-d-glucopyranoside (**17**) [35,36,37], five phenolic acid *viz* chlorogenic acid (**6**) [40], ellagic acid (**8**) [41], gallic (**11**), methyl gallate (**12**) [42], caffeic acid [40], (**18**) as well as two aglycones, kaempferol (**19**) and quercetin (**20**) [36]. All compounds were isolated from *C. viminalis* aerial parts for the first time except (**8**, **11**–**12**, **15**–**16**, and **20**). The structure of all compounds (Figure 1) was established using chemical and physical methods as 1-D and 2-D ^1^HNMR data as well as –ve HRESI MS data, in addition to comparison with authentic samples and previously reported data except for compounds (**6**, **8**, **11**, **12**, **14**, **18–20**), which were identified with –ve HRESI MS and comparison with authentic samples.

#### 2.1.1. Kaempferol 3-*O-*(4″-galloyl)-*β*-d-glucopyranosyl-(1‴→6″)-*O*-*β*-d-glucopyranoside (**1**)

**Figure 2:**
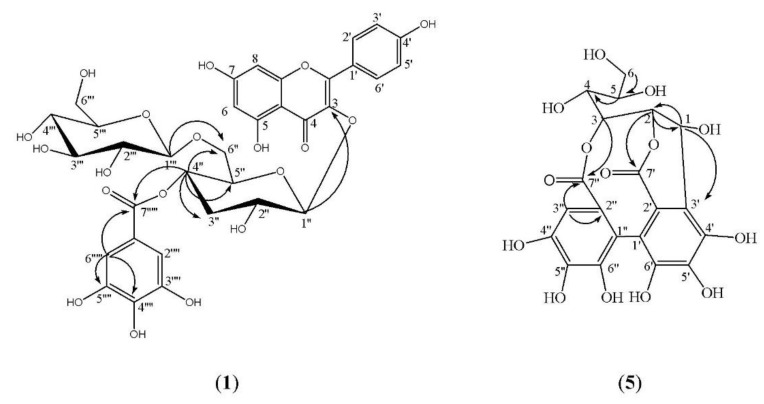
HMBC correlations of compound (**1**) and (**5**).

Compound **1** was isolated as a yellow amorphous powder and gave dark purple color under UV light on PC which changed to yellowish-green and green color upon spraying with Naturstoff and FeCl_3_, respectively. Moreover, it gave kaempferol and gallic in the organic phase and glucose in the aqueous one upon acid hydrolysis and comparison with authentic sample on PC (COPC). Therefore, based on its chromatographic data and acid hydrolysis it was expected that **1** is related to kaempferol 3-*O*-glycoside [37]. ^1^H NMR spectrum of **1** (Table 1) showed A_2_X_2_ spin coupling system of two *ortho-*doublets protons at δ_H_ 8.07 and 6.86 for H-2′/6′and H-3′/5′, respectively, together with an AM system of two *meta*-doublets protons for δ_H_ 6.44 and 6.21 for H-8 and H-6, respectively, which are characteristic for kaempferol aglycone [34]. This finding is further confirmed by their ^13^CNMR spectrum (Table 1) which displays fifteen carbon signals characteristic for kaempferol aglycone with the key signals at δ_C_ 177.8 (C-4), 131.4 (C-2′/6′), 115.7 (C-3′/5′and 160.3 (C-4′) [36]. Additionally, ^1^H NMR data displayed a singlet signal integrated for two protons at δ_H_ 6.97 characteristics for H-2′′′′/6′′′′ of galloyl moiety [43]. Moreover, the presence of two doublet signals in the aliphatic region at δ_H_ 5.39 and 5.46 (*J* = 8 Hz) gave evidence for the presence of two anomeric protons (H-1″and H-1‴) characteristic for glucose moiety which agreed with the acid hydrolysis results. The presence of galloyl moiety was confirmed by ^13^C NMR and HMBC data (Table 1, Figure 2), through the presence of seven carbons signals with the key signals among which δ_C_ 165.7 for the carbonyl carbon (C-7′′′′) and δ_C_ at 145.8 and 109.2 for C-3′′′′/5′′′′ and C-2′′′′/6′′′′, respectively as well as the HMBC correlation between H-2′′′′/6′′′′ and C-1′′′′ (δ_C_ 119.5), C-4′′′′ (δ_C_ 139.0), C-3′′′′/5′′′′(δ_C_ 145.9 ppm) and C-7′′′′ (δ_C_ 165.7 ppm). The two sugar moieties were established by the presence of twelve carbon signals, two of them are anomeric carbons at δ_C_ 102.1 (C-1″), and 101.2 (C-1‴) and the remaining carbon signals were at the range between δ_C_ 77.7-60.8, which indicated that the two-sugar moiety consisted of two β-d glucopyranosides [35,36]. The sugar moieties are attached to C-3 of the kaempferol aglycone due to the up-field shift of C-3 (δ_C_ 133.8) and downfield shift of C-2 at δ_C_ 156.8 [36], as well as the HMBC correlation between H-1″ (δ_H_ 5.39) and C-3 (δ_C_ 133.8). The inter glycosidic linkage between the two glucose units was 1‴→6″ due to the downfield shift of H-6″ in the ^1^H NMR at δ_H_ 3.66, corresponding to the free one δ_H_ 3.29 (C-6‴), in addition to the downfield shift of C-6″ at δ_C_ 68.2 vs. C-6‴ (δ_C_ 60.8). Additionally, the correlation between H-1‴ (δ_H_ 5.46) and C-6″ (δ_C_ 68.2) in the HMBC spectrum. The position of galloyl moiety was confirmed at C-4″ due to the downfield shift of H-4″ in the ^1^H NMR spectrum at δ_H_ 4.82 as well as its downfield in the ^13^C NMR at δ_C_ 71.9, in addition to the upfield shift of C- 3″ (δ_C_ 74.3) and C-5″ (δ_C_ 71.6). Moreover, the correlation between H-4″ (δ_H_ 4.82) and C-7′′′′ (δ_C_ 165.7), C- 3″ (δ_C_ 74.3), C-5″ (δ_C_ 71.6) and C-6″ (δ_C_ 68.2) in the HMBC. HMQC spectrum confirmed that all non-exchangeable proton resonances were associated with the directly attached carbon atoms. Therefore, compound **1** was identified as kaempferol 3-*O-*(4″-galloyl)-*β*-d-glucopyranosyl-(1‴→6″)-*O*-*β*-d-glucopyranoside, which was isolated for the first time from nature.

#### 2.1.2. Kaempferol 3-*O*-*β*-d-mannuronopyranoside (**2**) and kaempferol 3-*O*-*β*-d-mannopyranoside (**3**)

Compounds **2** and **3** were isolated as yellow amorphous powders and they gave the chromatographic properties characteristic for kaempferol 3-*O*-glycoside as in **1** [37]. Additionally, they gave kaempferol as aglycone (organic phase) and mannuronic acid in case of **2** and mannose in **3** (aqueous phase) upon acid hydrolysis and comparison with authentic sample and spraying reagents on PC. Therefore, based on their chromatographic data and acid hydrolysis it was expected that **2** and **3** are related to kaempferol 3-*O*-glycoside [36,37]. ^1^H and ^13^C NMR spectrum of **2** and **3** (Table 2) displayed the proton and carbon signals characteristic for kaempferol aglycone as in **1**. Concerning the sugar moiety, ^1^H NMR of both compounds showed a doublet singlet at δ_H_ ≈ 5.4 with small J-coupling (≈2.4 Hz) for H-1″ characteristic for mannuronic acid and mannose [44,45]. Moreover, from their ^13^C NMR data, the presence of mannuronic acid in **2** was established due to the presence of C-1″ at δ_C_ 101.6 and C-6″ at δ_C_ 171.5, together with four carbon signals ranged between δ_C_ 76.4–74.3, while mannose in case of **3** was confirmed by the presence of six carbon signals among which, C-1″ (δ_C_ 101.6) and C-6″ (δ_C_ 62.3). Therefore, the sugar moiety was established as *β*-d-mannuronopyranoside and *β*-d-mannopyranoside, in case of **2** and **3,** respectively [33], which agreed with the result of acid hydrolysis. The attachment of the sugar moieties was at C-3 of the aglycone, as in the case of **1** due to the up-field shift of C-3 at δ_C ≈_133.5 and downfield shift of C-2 at δ_C_ 156.8 [36]. Final confirmation of the compound **2** and **3** was established from their -ve HRESI-MS, which revealed the presence of a molecular ion peak at *m/z* 461.1359 [M − H]^−^ and 447.0941 [M − H]^−^ for kaempferol 3-*β*-d-mannuronopyranoside (**2**) kaempferol 3-*O*-*β*-d-mannopyranoside (**3**), which were also isolated for the first time from nature.

#### 2.1.3. Quercetin 3-*O*-*β*-d-mannuronopyranoside (**4**)

Compound **4** was isolated as an amorphous yellow powder, it appeared as a dark purple color under UV light, which was changed to orange and green color by spraying with Naturstoff and FeCl_3_, respectively. Therefore, based on these data, it was expected that **4** belonged to quercetin 3-*O*-glycoside [37]. Complete acid hydrolysis of **4** resulted in quercetin aglycone in the organic phase and mannuronic acid in the aqueous one (COPC). Further evidence for the structure of **4** resulted from ^1^H NMR data which showed two aromatic spin coupling systems, one is ABX system at δ_H_ 7.89, 7.48 and 6.84 for H-2′, H-6′ and H-5′, respectively and the other is AM system at δ_H_ 6.40 and 6.19 for H-8 and H-6, respectively supporting the presence of quercetin nucleus [37]. Moreover, the presence of doublet signal at δ_H_ 5.35 with small *J* coupling 2.0 Hz in the aliphatic region gave evidence for the presence of mannose or mannuronic acid as a sugar moiety [44,45]. Moreover, ^13^C NMR data displayed the fifteen carbon signals resonances characteristic for quercetin nucleus [36], in addition to six carbon signals characteristic for mannuronic acid among which C-1″ (δ_C_102.2) and C-7″ (δ_C_171.9) [46]. Final confirmation of **4** was established from -ve HRESI-MS data, which showed molecular ion peak at *m/z* 477.1472 with fragment ion peak at 301.1082 [quercetin−H]^–^. Accordingly, **4** was identified as quercetin 3-*O*-*β*-d-mannuronopyranoside, which was isolated for the first time from nature.

#### 2.1.4. 2,3 (*S*)-hexahydroxydiphenoyl-d-glucose (**5**)

Compound **5** showed a dark purple-fluorescent spot under short UV-light, which turned to an indigo-red and deep blue color by spraying with NaNO_2_, glacial AcOH and FeCl_3_, respectively characteristic for hexahydroxy diphenoyl (HHDP) containing ellagitannin. Moreover, it did not give ellagic acid or glucose on complete acid hydrolysis, which is considered as strong evidence for the *C-*glycosidic structure of **5**. ^1^H NMR data (Table 3) displayed one singlet signal at δ_H_ 6.16 for H-3″ of HHDP ester moieties with the loss of one proton (H-3′), due to the oxidative coupling and formation of extra C-C linkage with the anomeric position of the sugar moiety. Furthermore, the *C*-glycosidic structure and open-chain glucose moiety in the structure was in turn established from the appearance of sugar protons in the range of δ_H_ 5.43–3.34. Additionally, the appearance of the anomeric protons as a doublet with a small *J* value (4.8 Hz) at δ_H_ 5.42 was indicative of a *β* specific absolute configuration at C-1 with axial OH [47,48]. The position of attachment of HHDP moiety at H-2 and H-3 was established from the downfield shift of H-2 and 3 at δ_H_ 4.56 and 5.06, respectively. Further confirmation of the structure was obtained from ^13^CNMR which displayed the six carbons signal for glucose moiety in the range of 63.2- 76.5. The open-chain nature of glucose instead of hemiacetal ^4^C_1_–pyranose was established due to the strong up-field shift of C-1 at δ_C_ 66.9, in comparison to that of pyranose (δ ≈ 90–95 ppm). Likewise, the *C*-glycosidic nature was further evidenced by the typical downfield shift of C-3′ (HHDP) at 116.2 (≈ +10) and up-field location of C-7′ at 164.3 (≈-6) concerning those of C-3″ at 103.4 and C-7″ at 170.6 [34,35]. The assignment of the remaining carbon signal characteristic for open-chain sugar and HHDP moiety was compared with the reported data of *C*-glycosidic tannins [47,48,49]. HMQC spectrum confirmed that all non-exchangeable proton resonances were associated with the directly attached carbon atoms. Additional confirmation of the compound was established from HMBC (Table 3, Figure 2), which showed the correlation between H-1 (δ_H_ 5.43) with C-3′ (δ_C_ 116.2) and C-2 (δ_C_ 76.5), as well as the correlation between H-2 (δ_H_ 4.56), C-1 (δ_C_ 66.9) and C-7′ (δ_C_ 164.3), which confirmed the C-C linkage of **5.** Moreover, H-3″ (δ_H_ 6.16) was confirmed from the correlation with C-2″, C-7″. Final confirmation was established from the negative HRESI/MS, which displayed a molecular ion peak at *m/z* 481.0970 [M − H]^−^ corresponding to a mono-hexahydroxydiphenoyl-glucose. Accordingly, Compound **5** was identified as 2,3 (*S*)-hexahydroxydiphenoyl-d-glucose, which was isolated from nature for the first time.

### 2.2. Antioxidant Activity

Erkan et al., 2008 [50], reported the close correlation between radical scavenging activity, total flavonoid, and phenolic content of various natural sources. The scavenging ability of DPPH free radical is commonly used for evaluation of the antioxidant activity of natural compounds. Moreover, there are several in vitro complementary assays that are used based on the inactivation of NO and O_2_^−^ radicals [51]. In this study, different assays were used to evaluate the antioxidant potential of the extract and some pure compounds to establish the mechanism of action of them [52]. We noticed that AME and pure tested compounds **2**–**4**, **7**, **10**, and **15**–**17** exhibited strong antioxidant activity through their ability to inhibit DPPH, NO, and O_2_^−^ radicals (Table 4).

#### 2.2.1. DPPH Radical Scavenging Activity

This assay is based on the ability of the stable DPPH radical to react with hydrogen donors including phenolics. We found that all compounds exerted higher DPPH scavenging activity than that of ascorbic acid (IC_50_ = 111.3 μM), with compounds **2**, **3**, **7**, and **17** being the most active compounds (IC_50_ = 13.2, 19.9, 11.4 and 19.2 μM, respectively). While AME (IC_50_ = 25.48 μg/mL) exhibited close activity to that of ascorbic acid (19.63 ± 0.37 μg/mL).

#### 2.2.2. NO Radical Scavenging Activity

Nitric oxide (NO) is a vital chemical mediator generated by neurons, macrophages, and endothelial cells. It is involved in the regulation of various physiological processes. However, excess generation of NO is associated with numerous diseases [53]. Under aerobic conditions, NO reacts with oxygen to produce nitrite. Scavengers of NO compete with oxygen leading to reduced production of nitrite ions. The results showed that all compounds showed activity more than that of ascorbic acid (IC_50_ = 73.5 μM). The most active compound was compound **16** (IC_50_ = 17.4 μM) being four times more active than ascorbic acid, whereas AME (IC_50_ = 12.67 μg/mL) exhibited similar activity to that of Ascorbic acid (IC_50_ = 12.95 ± 0.77 μg/mL).

#### 2.2.3. SOD Radical Scavenging Activity

Superoxide anion radicals are the early radicals with basic role in the formation of ROS as previously reported [54]. Our results showed that AME exert the highest ability to inhibit O_2_^−^ radical with IC_50_ = 0.50 µg/mL, which is nearly twenty folds that of ascorbic acid (IC_50_ = 21.62 µg/mL). Furthermore, all pure compounds exhibited superior O_2_^−^ radical scavenging activity to that of ascorbic acid, (Table 4).

Several mechanisms have been suggested for the antioxidant activity exhibited by flavonoids being as: suppression of ROS formation, which could be through inhibition of enzymes or chelation of trace elements involved in the free radical generation; scavenging of ROS, or protection of antioxidant defense systems [54,55]. Moreover, some effects mediated by flavonoids may be owing to a combination between their radical scavenging activity and their ability to inhibit enzymes involved in ROS generation [56]. Furthermore, flavonoids act on cell signaling pathways, inhibit expression of pro-oxidative enzymes as microsomal monooxygenase, mitochondrial succinoxidase, NADH oxidase and lipoxygenases (LOX) as well as inducing expression of anti-oxidative enzymes (e.g., catalase, superoxide dismutase) [57].The antioxidant activity exhibited by flavonoids depends on several structural features like the arrangement of functional groups, the configuration and substitution, in addition to, the total number of hydroxyl groups, which significantly affect different antioxidant capability mechanisms as radical scavenging and metal ion chelation ability [58,59]. Correspondingly, the hydroxyl group configuration on the B ring; especially its catechol structure represents the most significant determinant for ROS scavenging as it can donate a hydrogen and an electron to peroxyl, hydroxyl, superoxide, and peroxynitrite radicals, stabilizing them and forming relatively stable flavonoids radical [58,59,60]. Additionally, different reports showed that flavonoids having an unsaturated 2-3 bond in conjugation with a 4-oxo function and the conjugation between the A and B rings also exhibited potent antioxidant activities. Our study showed that the nucleus of compounds identified from AME of *C. viminalis* was mainly based on quercetin and kaempferol nucleus, which have the above-mentioned structure features allowing them to exert a strong antioxidant activity as revealed in our results. Many reports mentioned the antioxidant activity of quercetin [19,61], and kaempferol [19]. Moreover, the AME showed to contain other compounds as gallic acid [62,63,64], methyl gallate [64,65,66], ellagitannins [67], caffeic acid [68], chlorogenic acid [69], and ellagic [70], which all possess strong antioxidant activity.

### 2.3. Cytotoxic Activities of AME and the Pure Isolated Compounds against MCF-7 and HepG2 Cell Lines

Cancer is considered as one of the top leading causes of death all over the world and according to the WHO (2015) [71] Breast cancer is one of the four major cancer types and it represents the highest incidence rate as well as the second-highest cause of death in females [71,72]. Additionally, hepatocellular cancer represents the sixth most frequent cancer and is the second leading cause of death [73]. The main difficulty in fighting cancer is the limited specificity of available chemical treatments. Thus, identifying new compounds with promising antitumor activity is extremely required, leading the researchers to seek for screening plants and isolation of natural compounds. Dietary flavonoids comprise many polyphenolic secondary metabolites with several pharmacological activities like their potential role as anti-cancer. A positive correlation between a diet rich in fruit and vegetables (and consequently rich in flavonoids) and lower risk of breast cancers, colon, and prostate cancers raised a question whether flavonoids can act as chemopreventive agents or can affect various genes and proteins to play role in chemotherapy [74]. There is a strong correlation between oxidative stress and the prevalence of cancer. Therefore, we investigated the anticancer activity of the AME and pure tested compounds **2**–**4**, **7**, **10**, and **15**–**17** against MCF-7 and HepG2 cell lines using the MTT assay (Table 5). It was found that compounds **4**, **7**, and **16**, showed strong anticancer activity against MCF-7 breast cancer cells (IC_50_ =127.4, 101.0, and 132.5 μM, respectively), followed by **3** and **15** which displayed moderate activity in comparison to other compounds. While most of the compounds showed greater cytotoxic effect in the case of HepG2 cell line. Compounds **3**, **7**, and **16** exhibited the strongest activity (IC_50_ = 90.0 μM for compound **7** and 100.2 μM for 3 & 16, respectively), whilst compounds 4, 15, and 17 showed strong to moderate activities with IC_50_ = 125.9, 132.5, and 126.1 μM, respectively. Thus, the above-mentioned compounds may be a potential new antitumor and/or adjuvant treatment against liver and breast cancer. Since ROS can induce malignant cell transformation, thus, inhibition of ROS production can reverse cancer cell phenotype. Conclusively, polyphenolic compounds as kaempferol [75], quercetin [76], and their glycosides, tannins [67], and phenolic acids [77] can control cancer through their antioxidative activity. Therefore, the anticancer activity of AME and the isolated phenolic compounds may be attributed to their antioxidant activity.

### 2.4. In Silico Analysis for the Antioxidant Effect

**Figure 3:**
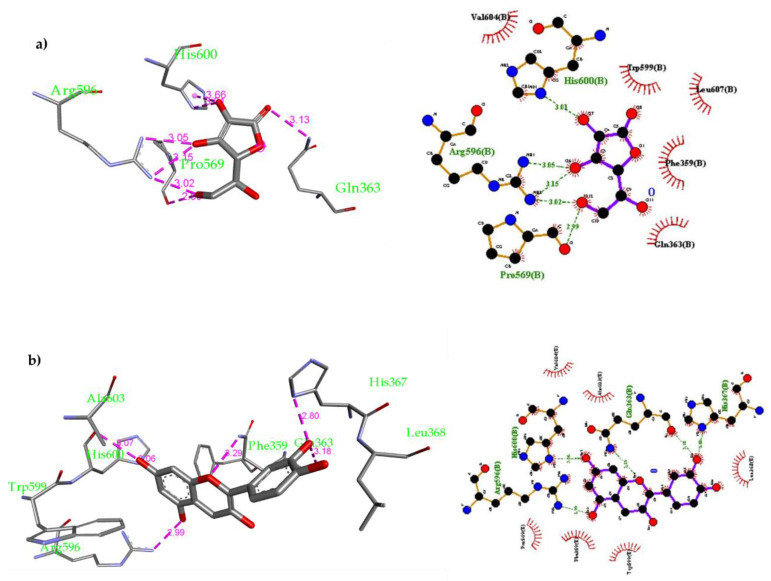
(**a**) Docking of Ascorbic acid inside the 5-LOX active site and 2-D schematic representation for the non-covalent interactions of ascorbic inside 5-LOX active site; (**b**) Docking of Quercetin inside the 5-LOX active site and 2-D schematic representation for non-covalent interactions formed by quercetin inside the 5-LOX active site.

The inhibition of 5-LOX could be a potential therapeutic pathway to treat and prevent cancer formation via inhibiting several oxidative and inflammatory processes. Recently, some crystal structures of human stable-5-LOX in bound to different natural products were resolved [78]. One of these is the crystal structure of nordihydroguaiaretic acid (NDGA) occupying the 5-LOX active site. NDGA is one of the plant polyphenols that has antioxidant properties and was found to inhibit 5-LOX. NDGA is believed to inhibit 5-LOX by binding into the 5-LOX active site and preventing the oxidative conversion process of Fe^2+^ to Fe^3+^ that activates the enzyme. Many natural and synthetic compounds with redox and non-redox activity can inhibit 5-LOX enzyme. Ascorbic acid is considered one of the most potent, naturally occurring, water-soluble antioxidant that has been widely studied for the treatment of several inflammatory conditions because of its strong antioxidant property [79]. In addition, it was found that quercetin is the most potent natural flavonoid against 5-LOX [80]. In this study, a thorough molecular docking inside the 5-LOX active site was performed on compounds **2**, **3**, **7**, **16**, **17**, ascorbic acid and quercetin. The molecular docking was performed by AutoDock Vina [81] using the crystal structure of human stable-5-LOX in bound to NGDA (PDB: 6N2W). Validation for the docking parameters was performed by re-docking NGDA inside the active site of 5-LOX. The binding mode was assessed by comparing the modeled structure’s binding mode to that of the crystal structure. It was found that the modeled structure of NDGA overlays on its crystal structure forming the same interactions (see Supplementary Materials). Reference drugs, i.e., ascorbic acid and quercetin, were also docked inside the 5-LOX binding site. The docking of ascorbic acid resulted in 9 conformers with affinity ranging from −4.6 to −4.9 kcal/mol. Ascorbic acid was found to fit nicely in the active site forming multiple H-bonds with proximal amino acid (Figure 3a): Gln363 (3.13 Å), Pro569 (2.99 Å), Arg595 (3.02 Å, 3.05 Å and 3.15 Å) and His600 (3.01 Å). Moreover, quercetin was docked as well inside the 5-LOX active site; 9 conformers were also obtained with binding affinity ranging from −6.0 to −7.2 kcal/mol. Quercetin interactions inside the 5-LOX active site are as follows: 1) H-bonds with Gln363 (3.18 Å), His367 (2.80 Å), Arg596 (2.99 Å) and His600 (3.06 Å and 3.07 Å); 2) Van der Waals interactions with Phe359, Trp599 and Ala603 (Figure 3b).

**Figure 4:**
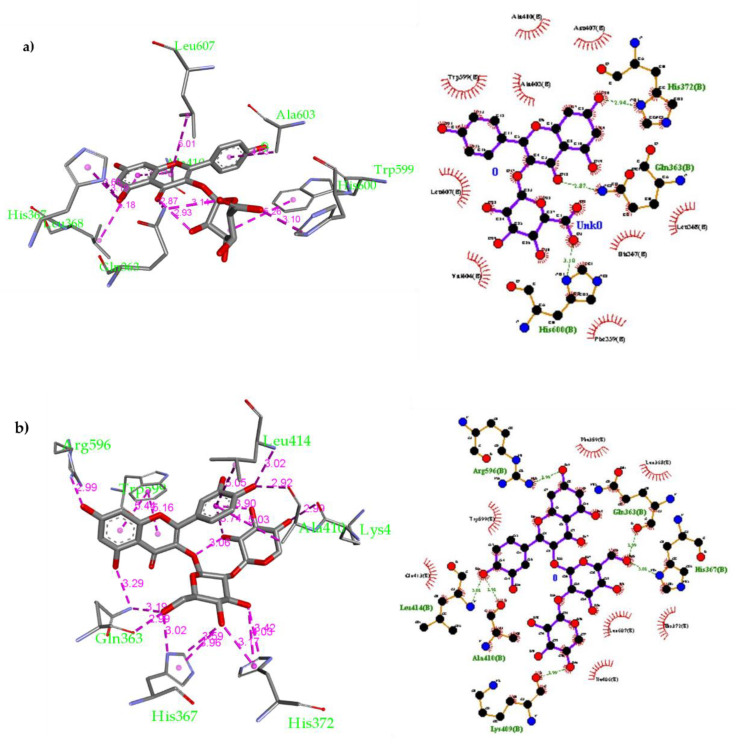
(**a**) Docking of compound **2** inside the 5-LOX active site and 2-D schematic representation for its non-covalent interactions inside 5-LOX active site; (**b**) Docking of compound **7** inside the 5-LOX active site and 2-D schematic representation for its non-covalent interactions inside the 5-LOX active site.

Compound **2** was docked inside the 5-LOX active site using the same parameters and 9 possible conformers were obtained with higher affinity than ascorbic acid ranging from −7.1 to −8.0 kcal/mol. Compound **2** was found to form several non-covalent interactions inside the 5-LOX active site (Figure 4a): (1) H-bonds with His367 (3.19 Å), Gln363 (2.87 Å and 3.14 Å) and His600 (3.10 Å); (2) Van der Waals interactions with Leu368, Ala410, Trp599 and Leu607. Additionally, Compound **3** was docked inside the 5-LOX and 9 conformers were obtained with moderate affinity ranging from −6.4 to −7.4 kcal/mol (see Supplementary Materials). Furthermore, compound **7** was also docked, and 9 conformers were obtained with better affinity ranging from −6.9 to −8.1 kcal/mol. Several non-covalent interactions were formed between compound **7** and the 5-LOX amino residues (Figure 4b): (1) multiple H-bonds with Gln363 (2.70 Å), His367 (3.02 and 3.59 Å) and Leu414 (3.02 Å); (2) Van der Waals interactions with Ala410, Leu414 and Trp599. Compound **16** was docked inside the 5-LOX active site and 9 conformers were obtained with strong affinity ranging from −7.2 to −8.3 kcal/mol. This compound was found to have the best fitting among all compounds docked.

**Figure 5:**
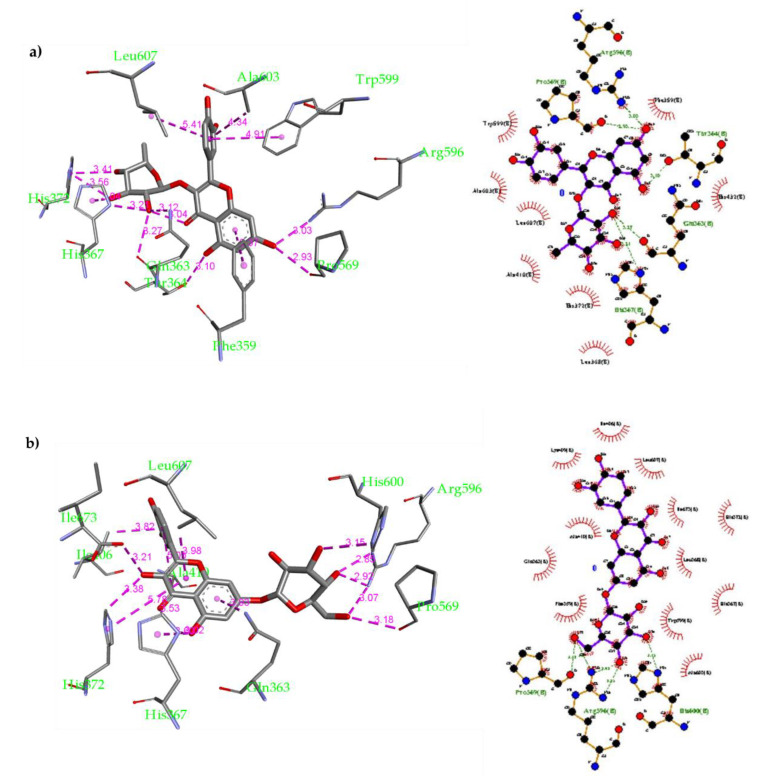
(**a**) Docking of compound **16** inside the 5-LOX active site and 2-D schematic representation for its non-covalent interactions inside 5-LOX active site; (**b**) Docking of compound **17** inside the 5-LOX active site and 2-D schematic representation for its non-covalent interactions inside the 5-LOX active site.

Compound **16** was found to form more non-covalent interactions than others (Figure 5b): (1) H-bonds with Gln363 (3.05 and 3.12 Å), Thr364 (2.81 Å), His367 (3.12 Å) and Pro569 (2.51 Å), Arg596 (3.03 Å); (2) Van der Waals interactions with Phe359, Trp599, Ala603 and Leu607. In addition, compound **17** was docked as well and 9 conformers were obtained with affinity ranging from −6.9 to −8.1 kcal/mol. This compound was found to have good fitting inside the 5-LOX active site. Compound **17** was found to form several non-covalent interactions (Figure 5b): (1) H-bonds with His367 (2.64 Å), Arg596 (3.07 and 3.38 Å) and His600 (3.13 Å); (2) Van der Waals interactions with His372, Ile406, Ala410 and Leu607. The docking results suggest that all the six polyphenolic compounds are well-fitted inside the human 5-LOX active site and exert binding affinity greater (more favorable) than ascorbic acid and quercetin. It is expected that these compounds will exhibit a good in vitro profile for inhibiting human 5-LOX through occupying its active site preventing the conversion of Fe^2+^ to Fe^3+^ that activates the enzyme. Additionally, compound **17** has a good chance to further in vivo use as it could survive through human digestion, get absorbed then inhibit human 5-LOX at its site of action.

## 3. Materials and Methods

### 3.1. General Experimental Data

The NMR data were recorded on Bruker Avance spectrometers (400 and 500 for ^1^H NMR and 100 and 125 MHz for 13C NMR). The results were expressed as δ ppm values relative to the internal reference (TMS). ESI-MS was done on a Xevo TQD Triple quadrupole (Water Corporation, Milford, USA). Column chromatography (CC) was carried out using Polyamide S (Fluka Chemie AG, Buchs, Switzerland), Sephadex LH-20 (Sigma-Aldrich, Steinheim, Germany), and microcrystalline cellulose (Merck, Darmstadt, Germany). Whatman No.1 paper sheets (Whatman Ltd., Maidstone, Kent, UK), silica gel F254, and cellulose plates (0.2 mm, Merck, Darmstadt, Germany) were used for qualitative identification of the fractions and pure compounds.

*n*-Butanol: acetic acid: water, [4:1:5 (BAW), top layer, S_1_], 15% acetic acid (S_2_) and n-butanol: isopropyl: water, [4:1:5 (BIW), top layer, S_3_] were used for elution of compounds on a thin layer (TLC), paper (PC) and column chromatography. Naturstoff [82], FeCl_3_, NH_3_, NaNO_2_ [83], glacial AcOH, and aniline hydrogen phthalate [84] are representing the spray reagents used for the visualization of the pure compounds.

Authentic samples of flavonoids (kaempferol 3-*O*-*β*-d-rhamnopyranoside, kaempferol and quercetin), phenolic acids (chlorogenic acid, ellagic acid, gallic, methyl gallate, and caffeic acid), and sugars (purity 95%) were obtained from the Pharmacognosy Department, Faculty of Pharmacy, Helwan University, Helwan, Cairo, Egypt. Dichloromethane, ethanol, ethyl acetate, methanol, butanol, petroleum ether and acetic acid, dimethyl sulfoxide (DMSO) are of analytical grade and obtained from El Nasr Pharmaceutical Chemicals Co (Cairo, Egypt).

2,2-diphenyl-1-picrylhydrazyl radical (DPPH), pyrogallol, sodium nitroprusside, Griess reagent (1% of sulphanilamide and 0.1% of naphthyl ethylenediamine in 2.5% H_3_PO_3_), ascorbic acid and MTT (3-[4, 5-dimethylthiazol-2-yl]-2, 5-diphenyl tetrazolium bromide) were purchased from Sigma-Aldrich (Steinheim, Germany). Fetal bovine serum, RPMI 1640 medium, HEPES buffer solution, L-glutamine, gentamycin, and 0.25% Trypsin-EDTA were supplied from Lonza bioscience (Basel, Switzerland). Human cancer cell lines: breast cancer cell lines (MCF-7) and hepatocellular carcinoma cell lines (HepG-2) were obtained from VACSERA Tissue Culture Unit (Giza, Egypt).

### 3.2. Plant Material

*Callistemon viminalis* (Sol. ex Gaertn.) G.Don. (Syn, *Melaleuca viminalis* (Sol. ex Gaertn. *Metrosideros viminalis* (Sol. ex Gaertn.) Byrnes. The aerial parts were collected from El Qanater El Khayreya, Qalyubia, Egypt during the flowering stage (December 2016). The plant was taxonomically identified by Dr. Trease Labib, former specialist of plant Taxonomy at El Orman Botanical Garden, Giza, Egypt. Voucher specimens (16 Mvi1/2016) were placed in the Pharmacognosy Department Herbarium, Faculty of Pharmacy, Helwan University, Cairo, Egypt.

### 3.3. Extraction and Isolation

Aerial parts of *C. viminalis* (1.7 kg) were dried, ground to a small size, and then extracted with 80% aqueous methanol (3 × 5 L) giving 442.5 g dry extract after solvent evaporation under vacuum. The obtained dry extract was defatted with petroleum ether (60–80 °C), followed by evaporation of solvents to give 150.5 and 275.5 g of petroleum ether and aqueous residue, respectively. For purification of the extract from sugar and salts, the dried aqueous extract (275.5 g) was dissolved in the least amount of water and precipitated with excess ethanol (1:10 *v/v*), after filtration the ethanol-soluble portion was evaporated leaving 175 g dry crude extract. The dried crude extract was found to be rich in polyphenolics based on its 2-D–PC using S_1_ and S_2_ systems and visualization under UV light and spraying with Naturstoff, NH_3_, NaNO_2_-glacial AcOH, and FeCl_3_ reagents. For isolation of the compounds 100 g from AME, it was subjected to fractionation on polyamide column (300 g polyamide,120 × 5 cm) using gradient elution with H_2_O-MeOH mixtures (100:0–0:100%) to obtain 105 individual fractions (each 1 L). They were collected into ten collective fractions (I-X), depending on their chromatographic behavior on TLC and PC using UV light and different spraying reagents. Fraction 1 (100% H_2_O, 10 g contained traces of phenolic compounds. Fraction II (10% MeOH: H_2_O, 6 g) was fractionated on a Sephadex LH-20 column and eluted with S_3_ to yield three subfractions (i, ii, iii). First, subfraction was subjected to repeated Sephadex LH-20 column using S_3_, followed by 50% MeOH: H_2_O to give a pure sample of **1** (19 mg) and 6 (5 mg). The second subfraction was applied on Sephadex LH-20 using S_3_ yielding **2** (20 mg) and **7** (25 mg) while the last subfraction revealed a pure sample of **8** (10 mg) by precipitation from its methanol solution. Fraction III (20–30% MeOH: H_2_O, 4 g) was subjected to fractionation on cellulose column and S_3_ as eluent resulted in two subfractions, the first was applied on Sephadex LH-20 and S_3_ as eluent to yield a chromatographically pure sample of **9** (12 mg) and **10** (15 mg). Moreover, compounds **11** (5 mg) and **12** (5mg) were purified from the second subfraction using PPC and S_2_ as eluent. Fractionation of IV (40% MeOH: H_2_O, 3.5 g) on cellulose column and elution with S_3_ resulted in two subfractions, the first gave a pure sample of **3** (20 mg) after application on Sephadex column (MeOH: H_2_O, 10–100%), while the second was subjected to repeated Sephadex columns and elution with S_3_, followed by MeOH: H_2_O (50%) to yield a pure sample of **4** (22 mg) and **13** (8 mg). Fraction V (40% MeOH: H_2_O, 3.0 g) was applied on repeated cellulose and Sephadex columns using S_3_ as eluent which revealed a chromatographically pure sample of **14** (15 mg) and **15** (10 mg). Compounds **5** (15 mg) and 15 (25 mg) were isolated from fraction VI (50–60% MeOH: H2O, 2.5 g) after application on cellulose column and S_3_ for elution, followed by repeated Sephadex columns using S_3_ and MeOH: H_2_O (50%) as eluents. Fraction VII (60–70% MeOH: H_2_O, 2.0 g) was subjected to fractionation on cellulose column (S_3_), then Sephadex column (S_3_) to give a chromatographically pure sample of **17** (15 mg) and **18** (5 mg). Fractions VIII (3 mg) and IX (2 mg) contained a complicated mixture and so remained under investigation. A pure sample of **19** (5 mg) and **20** (3 mg) were isolated from fraction X (100% MeOH: H_2_O, 1.5 g) by PPC using S_2_ for elution.

*Kaempferol 3-*O*-(4″-galloyl)-*β*-d-glucopyranosyl-(1‴→6″)-*O*-*β*-d-glucopyranoside* (**1**). The pure isolated compound is a yellow amorphous powder with Rf value S_1_ (0.60) and S_2_ (0.48). ^1^H and ^13^C NMR data (400 and 100 MHz, DMSO-*d*_6_) are presented in Table 1, Supplementary Materials Figures S1–S4.

*Kaempferol 3-*O*-*β*-d-mannuronopyranoside* (**2**). It was purified as a yellow amorphous powder with Rf value S_1_ (0.44) and S_2_ (0.47); Negative HRESI MS *m/z* 461.1359 [M − H]^−^calculated for C_21_H_18_O_12_, 923.2622 [2M − H]^−^, 285.0885[M-176; kaempferol]^−^. ^1^H and ^13^C NMR (400 and 100 MHz, DMSO-*d*_6_), Table 2, Supplementary Materials Figures S5–S7.

*Kaempferol 3-*O*-*β*-d-mannopyranoside* (**3**). It was isolated as a yellow amorphous powder; Rf; S_1_ (0.55) and S_2_ (0.49) Negative HRESIMS *m*/*z* 447.0941 [M−H]^−^, calculated for C_21_H_20_O_11_, 895.3108 [2M + H]^–^, ^1^H and ^13^C NMR data (400 and 100 MHz, DMSO-*d*_6_) are presented in Table 2, Supplementary Materials Figures S8–S10.

*Quercetin 3-O-β-*d*-mannuronopyranoside* (**4**). Yellow amorphous powder; Rf value S_1_(0.76); S_2_ (0.52); ^1^H NMR (400 MHz, DMSO-*d*_6_) δ 12.42 (OH-5), 7.48 (dd, br s, *J* = 7.2 Hz, H-6′), 7.89 (br s, H-2′), 6.84 (d, *J* = 7.2, H-5′), 6.40 (br s, H-8), 6.19 (br s, H-6), 5.35 (d, *J* = 2.0 H-1″), 3.59–3.27 (remaining sugar protons). ^13^C NMR (100 MHz, DMSO-*d*_6_), δ 177.8 (C-4), 171. 9 (C-6″), 164.9 (C-7), 161. 5 (C-5), 157.2 (C-2), 156.8 (C-9), 148.9 (C-4′), 145.3 (C-3′), 133.9 (C-3), 122.8 (C-6′), 121.9 (C-1′), 117.2(C-2′), 115.8 (C-5′), 104.2 (C-10), 102.2 (C-1″), 99.3 (C-6), 94.2 (C-8), 76.7 (C-5″),74.3 (C-3″), 74.3 C-2″), 71.9 (C-4″), Negative HRESIMS *m*/*z* 477.1472 [M − H]^−^ (calculated for C_21_H_18_O_13_, 477.1472), 955.2128 [2M − H]^−^, 301.1082 [M-176; aglycone]^−^, Supplementary Materials Figures S11–S13.

*2,3 (S)-hexahydroxydiphenoyl]-*d*-glucose* (**5**). It was isolated as off-white powder; Rf value S_1_ (0.03), S_2_ (0.44); Negative HRESIMS *m*/*z* 481.0970 [M−H]^−^ (calculated for C_20_H_18_O_14_, 481.0970). ^1^H and ^13^C NMR (400 and 100 MHz, DMSO-*d*_6_) refer to Table 3 Supplementary Materials Figures S14–S18.

### 3.4. In Vitro Antioxidant Assays

#### 3.4.1. 2,2-Diphenyl-1-picrylhydrazyl (DPPH) Radical Scavenging Activity

It was performed according to the method reported by [85]. In Brief, 100 μL of serially diluted (12.5, 25, 50, 100, and 200 µg/mL) AME and pure compounds, ascorbic acid and DMSO as a positive and negative control, respectively were added to 100 μL methanolic solution of DPPH. After vigorous shaking, the mixture was placed in the dark (25 °C, 30 min) followed by absorbance determination at λ517 nm. The experiment was performed in three replicates. The antioxidant activity was expressed as DPPH inhibition percentage (I%) as follows:

I% = [(Abs _control_-Abs _sample_)/Abs _control_] × 100, where Abs _control_ and Abs _sample_ are the control and sample absorbance, respectively. IC_50_ was calculated using nonlinear regression analysis from the curves plotted between the percentage of DPPH inhibition versus log concentration of sample or ascorbic acid.

#### 3.4.2. Antioxidant Capacity Determined by Nitric Oxide (NO) Scavenging Assay

The scavenging activity of the AME and pure compounds against nitric oxide was evaluated according to Ebrahimzadeh et al. [86], using ascorbic acid as a reference standard. 50 µL of different concentrations of the tested samples were pipetted into a 96-well plate followed by the addition of sodium nitroprusside (50 µL of 10 mM in PBS, pH 7.4) to each well. The plates were incubated for 90 min at room temperature, followed by the addition of an equal volume of Griess reagent. The absorbance of the developed color was measured at λ 546 nm. All measurements were performed in triplicates. The percentage of NO inhibition and IC_50_ were calculated as in the case of the DPPH method.

#### 3.4.3. Antioxidant Capacity Determined by Superoxide Radical (O_2_^−^) Scavenging Assay

Measurement of superoxide radical scavenging capacity of AME and the pure compounds was done using a previously reported method [87]. Ascorbic acid and DMSO were used as the positive and negative control, respectively. In Brief, 50 µL of each sample concentration were added to 2900 µL of 5 mM Tris HCl buffer (0.05 M, pH 7.4) containing 1 mM Na_2_EDTA. Then, 50 µL of 60 mM pyrogallol in 1mM HCl was added to the mixture. The absorbance of the reaction mixture was measured every 30 s for 5 min at λ325 nm. All measurements were performed in triplicates and the percentage of O_2_^−^ inhibition and IC_50_ were calculated as in the case of the previous antioxidant assay methods.

### 3.5. In Vitro Cytotoxic Activity

Cytotoxic activities of the AME and pure compounds were tested against Human Breast (MCF-7) and Hepatocellular carcinoma (HepG2) cancer cell lines using the microculture tetrazolium (MTT) assay and compared to that of untreated controls [88]. The cells were propagated in Dulbecco’s modified Eagle’s medium (DMEM) supplemented with 10% heat-inactivated fetal bovine serum, 1% L-glutamine, HEPES buffer, and 50 µg/mL gentamycin. All cells were maintained at 37 °C in a humidified atmosphere with 5% CO_2_ and were sub-cultured two times a week. For cytotoxicity assay, cells (1 × 10^4^ cells/ well), in the growth medium, were seeded in a 96-well microplate and treated with tested pure compounds and the extract (100 µl medium/well) at different concentrations (500, 250, 125, 62.5, 31.25, 15.6, 7.8 and 3.9 µg/mL in DMSO), then incubated for 24 h at 37 °C, in a humidified 5% CO_2_ atmosphere. After incubation, media were removed and 40 µL MTT solution/well was added and incubated for an additional 4 h. MTT crystals were solubilized by the addition of 180 µL of DMSO to each well and left at room temperature for 45 s. The presence of viable cells was visualized by the development of purple color due to the formation of formazan crystals. The absorbance was measured at λ 570 nm using a microplate reader (Sunrise, TECAN, Inc., CA, USA). Triplicate repeats were performed for each concentration. Data were expressed as the percentage of relative viability compared with the untreated cells and with the vehicle control (DMSO), with cytotoxicity indicated by <100% relative viability. The relation between surviving cells and drug concentration is plotted to get the survival curve of each tumor cell line after treatment with the specified compound. The half-maximal inhibitory concentration of viability (IC_50_) was calculated from the dose–response curve using nonlinear regression analysis.

### 3.6. Data Analysis

IC_50_ was calculated by using nonlinear regression analysis using Graph Pad Prism version 5 for windows (Graph Pad Inc., CA, USA). All experiments were carried out in triplicate and data were presented as mean ± SEM, using Graph Pad InStat (Graph software Inc., V 3.05, Ralph Stahlman, Purdue University, Lafayette, IN, USA).

### 3.7. Molecular Modeling Procedure

The crystal structure of the human stable 5-LOX (PDB: 6N2W) co-crystallized with NDGA (PDB ID: 1M17) was used for the docking analysis. 3D structures of Polyphenolic compounds as well as reference drugs (ascorbic acid and quercetin) were obtained using the Discovery Studio software (Accelrys Inc., San Diego, CA, USA). Auto Dock Tools (The Scripps Research Institute, La Jolla, CA, USA) was used to prepare the ligands and receptor as pdbqt files after removing water, adding polar hydrogen atoms and Gasteiger charges, respectively. The docking grid box size used was 40 × 40 × 40 Å^3^, encompassing the entire 5-LOX binding pocket. An exhaustiveness value of 8 was used while keeping the other parameters with their default values. The best docking pose (most stable) was selected for binding mode comparison with that of reference drugs. Visualization of ligand-protein non-covalent interactions was performed using Discovery Studio software. The schematic 2-D representations of enzyme-ligand complexes was generated using LIGPLOT version 2.2.4 (European Molecular Biology Laboratory, Cambridge, UK).

## 4. Conclusions

Natural antioxidants have several important uses in health promotion through inhibition of oxidative stress, which is an important character of any medicinal plant. The results of this study revealed that AME of *C. viminalis* aerial parts afforded four new flavonol glycosides and one tannin compound along with fifteen known compounds mainly flavonol glycosides, phenolic acids, and aglycones. The antioxidant activity of the AME and some isolated pure compounds was investigated, and we found that most of the isolated compounds showed significant radical scavenging activities using different radical scavenging assays, which merit additional studies of their cytotoxic activities against breast (MCF-7) and hepatocellular (HepG2) cancer cells. In addition, a thorough in silico analysis for possible antioxidant mechanism of action of these compounds was performed using the crystal structure of human 5-LOX. Six compounds were docked into the active site and the binding affinity was compared to that of ascorbic acid and quercetin. All compounds showed proper binding inside the 5-LOX active site exerting more favorable binding than reference drugs. The in silico experiments suggest that all compounds herein will have a good in vitro 5-LOX inhibition profile with some compounds, i.e., compound **17** have the potential for further clinical development. The docking studies, in vitro antioxidant and anticancer evaluations suggest that these compounds could be used as dual antioxidant and anticancer agents.

## Figures and Tables

**Table 1:** ^1^H (400 MHz) and ^13^C-NMR (100 MHz) in (DMSO) data for compound **1**.

Carbon No	δ _C_	δ_H_ *	H-MBC
**2**	156.8		
**3**	133.8		
**4**	177.8		
**5**	161.8		
**6**	98.9	6.21 (br s)	C-5, C-8, C-10
**7**	164.6		
**8**	94.2	6.44 (br s)	C-6, C-7, C-9, C-10
**9**	156.9		
**10**	104.4		
**1′**	121.2		
**2′/6′**	131.4	8.07 (d, 8 Hz)	C-2, C-4′
**4′**	160.3		
**3′/5′**	115.7	6.86 (d, 8)	C-1′, C-4′
**OH-5**		12.62	
**1″**	102.1	5.39 (d, 8)	C-3
**2″**	74.2 #	3.52	
**3″**	74.3	3.14	
**4″**	71.9	4.82	C-3″, C-5″, C-6″, C-7′′′′
**5″**	71.6	3.53	
**6″**	68.2	3.66	C-1‴
**1** **‴**	101.2	5.46 (d, 8)	C-6″
**2** **‴**	73.5 #	3.37	
**3** **‴**	77.7	3.08	
**4** **‴**	70.2	3.08	
**5** **‴**	76.1	3.33	
**6** **‴**	60.8	3.29, 3.44	
**1′′′′**	119.5		
**2′′′′/6′′′′**	109.2	6.97 (br s)	C_1′′′′,_ C_4′′′′,_ C_3′′′′/5′′′′′,_ C_7′′′′_
**3′′′′/5′′′′**	145.8		
**4′′′′**	139.0		
**7′′′′**	165.7		

Value between parentheses represent J value in Hz; * Value of sugar proton obtained from HMQC # Value may be interchangeable.

**Table 2:** ^1^H (400 MHz) and ^13^C-NMR (100 MHz) in (DMSO) data for compound **2** and **3**.

Carbon No	2		3	
	δ C	δ H	δ C	δH
**1**				
**2**	156.8		156.8	
**3**	133.5		133.9	
**4**	177.5		177.8	
**5**	161.5		161.5	
**6**	99.4	6.16 (brs)	97.5	6.14 (brs)
**7**	164.9		164.6	
**8**	94.2	6.38(brs)	94.4	6.36 (brs)
**9**	156.8		156.8	
**10**	104.1		104.4	
**1′**	121.1		121.4	
**2′/6′**	131.7	8.03 (d, 7.2)	131.7	8.03 (d, 6.8)
**4′**	160.6		160.3	
**3′/5′**	115.6	6.86 (d, 7.2)	115.7	6.86 (d, 6.8)
**OH-5**		12.48		12.49
**1″**	101.6	5.45(2.8)	101.6	5.43 (d, 2.4)
**2″**	74.3	4.14–3.25	72.1	3.51–3.24
**3″**	75.6		74.3	
**4″**	72.1		70.2	
**5″**	76.4		76.5	
**6″**	171.6		62.4	

Values between parentheses represent the *J* values in Hz.

**Table 3:** ^1^H (400 MHz) and ^13^C-NMR (100 MHz) in (DMSO) data for compound **5**.

Carbon No	δ _C_	δ_H_	HMBC	Carbon No	δ _C_	δ_H_	HMBC
**1**	66.9	5.43(d,4.8)	C-2, C-3′	5′	137.9		
**2**	76.5	4.56 (dd, 2, 4)	C-1, C-7′	6′	145.9		
**3**	71.4	5.06 (m)	C-7″	7′	164.3		
**4**	71.1	3.36(m)		1″	115.7		
**5**	74.6	3.57(dd, 3.2, 1.6)	C-4	2″	126.9		
**6**	63.2	3.34–3.44 (m)	C-5	3″	103.4	6.16(s)	C-2″, C-7″
**1** **′**	115.8			4″	144.3		
**2** **′**	119.9			5″	133.9		
**3** **′**	116.2			6″	145.3		
**4** **′**	143.0			7″	170.6		

Values between parentheses represent the *J* values in Hz.

**Table 4:** Antioxidant activities of the AME of the *C*. *viminalis* and pure compounds.

Compound/Extract	IC_50_ μg/mL ± SEM (IC_50_ μM)		
	DPPH	NO	SOD
**AME**	25.48 ± 0.29	12.67 ± 0.84	0.50 ± 0.29
**2**	6.08 ± 0.05 (13.2)	10.70 ± 1.58 (23.2)	10.18 ± 0.49 (22.1)
**3**	8.93 ± 0.17 (19.9)	11.21 ± 0.47 (25)	6.05 ± 0.57 (13.6)
**4**	15.86 ± 0.21 (33.3)	16.55 ± 0.29 (34.7)	6.00 ± 0.34 (12.6)
**7**	6.51 ± 0.18 (11.4)	15.10 ± 1.56 (26.5)	11.58 ± 0.41 (20.4)
**10**	18.13 ± 0.38 (39.2)	12.03 ± 0.76 (26.0)	6.76 ± 0.16 (14.7)
**15**	22.14 ± 0.31 (47.6)	11.51 ± 1.91 (24.8)	6.06 ± 0.57 (13.1)
**16**	19.39 ± 0.58 (43.3)	7.84 ± 0.88 (17.4)	21.48 ± 0.57 (48)
**1 7**	8.94 ± 0.28 (19.2)	13.13 ± 0.74 (28.2)	5.27 ± 0.49 (11.4)
**Ascorbic acid**	19.63 ± 0.37 (111.3)	12.95 ± 0.77 (73.5)	21.62 ± 0.40 (122.6)

Data are displayed as mean ± SEM of IC_50_ of three experiments. Value between () is IC_50_ in µM.

**Table 5:** The anti-proliferative effect of the AME of the *C*. *viminalis* and pure compound against MCF-7 and HepG2 cell lines.

Compound/Extract	IC_50_ μg/mL ± SEM (IC_50_ μM)	
	MCF-7 Cell Line	HepG2 Cell Line
**AME**	229.0 ± 5.2	214.0 ± 5.8
**2**	224.0 ± 4.6 (484.9)	175.0 ± 5.4 (378.8)
**3**	85.1 ± 3.1(190.0)	44.9 ± 2.8 (100.2)
**4**	60.9 ± 1.8 (127.4)	60.2 ± 2.7 (125.9)
**7**	62.0 ± 1.9 (101.0)	53.5 ± 3.1 (90.0)
**10**	124.0 ± 2.9 (268.4)	142.0 ± 5.2 (307.4)
**15**	86.4 ± 2.7 (186.2)	61.5 ± 2.9 (132.5)
**16**	59.4 ± 2.4 (132.5)	44.9 ± 2.4 (100.2)
**17**	101.0 ± 3.1 (217.7)	58.5 ± 2.7 (126.1)

Data are displayed as mean ± SEM of IC_50_ of three experiments. Value between () is IC_50_ in µM.

## Data Availability

The data presented in this study are available on request from the corresponding author.

## Supplementary Materials

The following are available online. Supplementary data includes molecular modeling, as well as spectral data.

## Sample Availability

Samples of the known compounds are available from the authors.

## Abbreviations

HCl	Hydrochloric acid;
HepG2	Hepatoma G2 cell line;
IC_50_	Concentrations of tested compounds that give about 50% inhibition of cell viability
MCF-7	Michigan Cancer Foundation-7 cell line;
MS	Mass Spectrometry;
NMR	Nuclear Magnetic Resonance;
