# Supplementary material for: Polyphenolic Profile of Callistemon viminalis Aerial Parts: Antioxidant, Anticancer and In Silico 5-LOX Inhibitory Evaluations
Source: Molecules. 2021 Apr 24;26(9):2481. doi: 10.3390/molecules26092481 (PMC8123052; doi:10.3390/molecules26092481)
Supplement: Supplementary file 1 [file molecules-26-02481-s001.zip › molecules-1190215-supplementary.pdf]

Polyphenolic Profile of *Callistemon viminalis* Aerial Parts: Antioxidant, Anticancer and *In Silico* 5-LOX Inhibitory Evaluations

Shahenda Mahgoub[1*], Nashwa Hashad[2], Sahar Ali[1], Reham Ibrahim[2], Ahmed M. Said [3,4*]
Fatma A. Moharram[2*], and Mohamed Mady [2]

[1]Biochemistry and Molecular Biology Department, Faculty of Pharmacy, Helwan University, Ein-Helwan, Helwan, Cairo 11795, Egypt

[2]Department of Pharmacognosy, Faculty of Pharmacy, Helwan University. Ein Helwan, Cairo, 11795, Egypt

[3]Department of Pharmaceutical Organic Chemistry, Faculty of Pharmacy, Helwan University, Ein-Helwan, Helwan, Cairo 11795, Egypt

[4] Department of Chemistry, University at Buffalo, The State University of New York, Buffalo, NY 14260, USA

*Correspondence: Fatma A. Moharram, Email: famoharram1@hotmail.com, Tel.: +202-2554-160; Ahmed M. Said, Email: ahmedmoh@buffalo.edu; Tel.: +1716-907-5016; Shahenda Mahgoub, shahenda.mahgoub@pharm.helwan.edu.eg

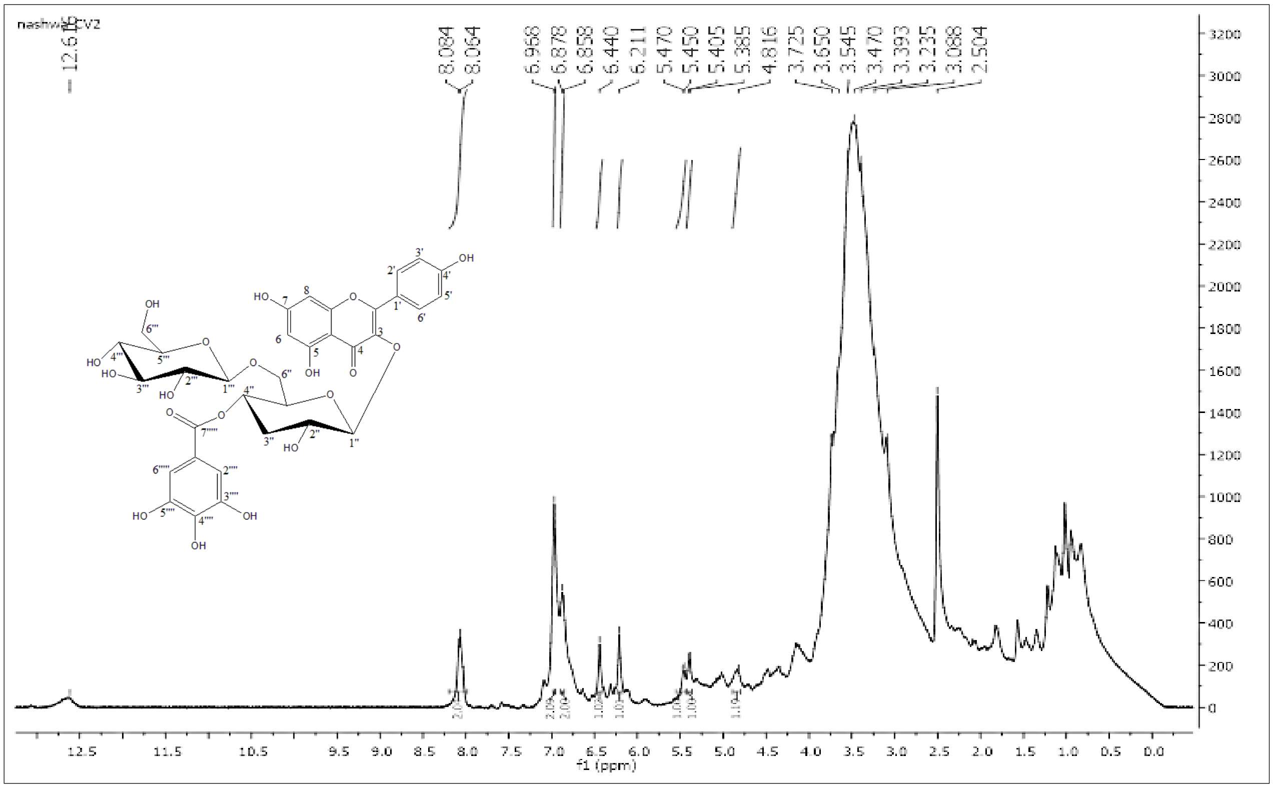

Figure S1: ^{1}HNMR spectrum of compound **1** (400 MHz, DMSO-d_6)

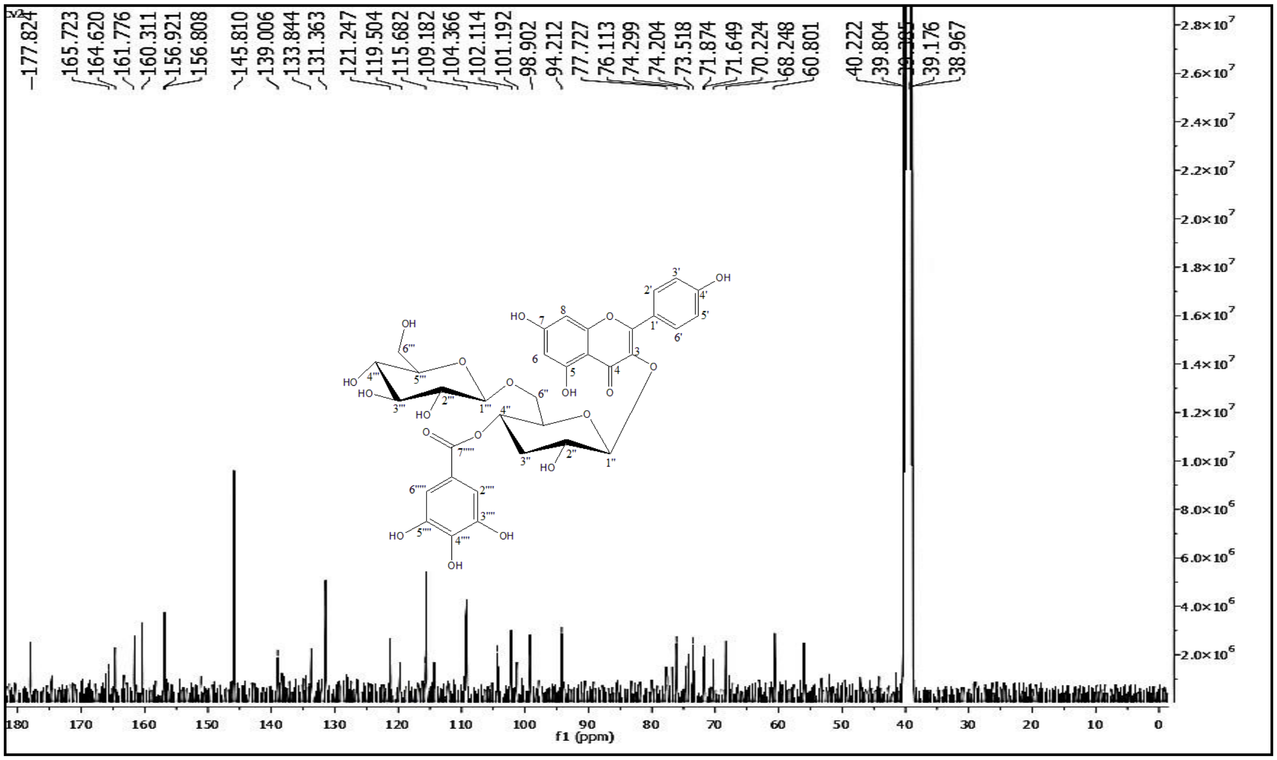

Figure S2: ^{13}CNMR spectrum of compound **1** (400 MHz, DMSO-d_6)

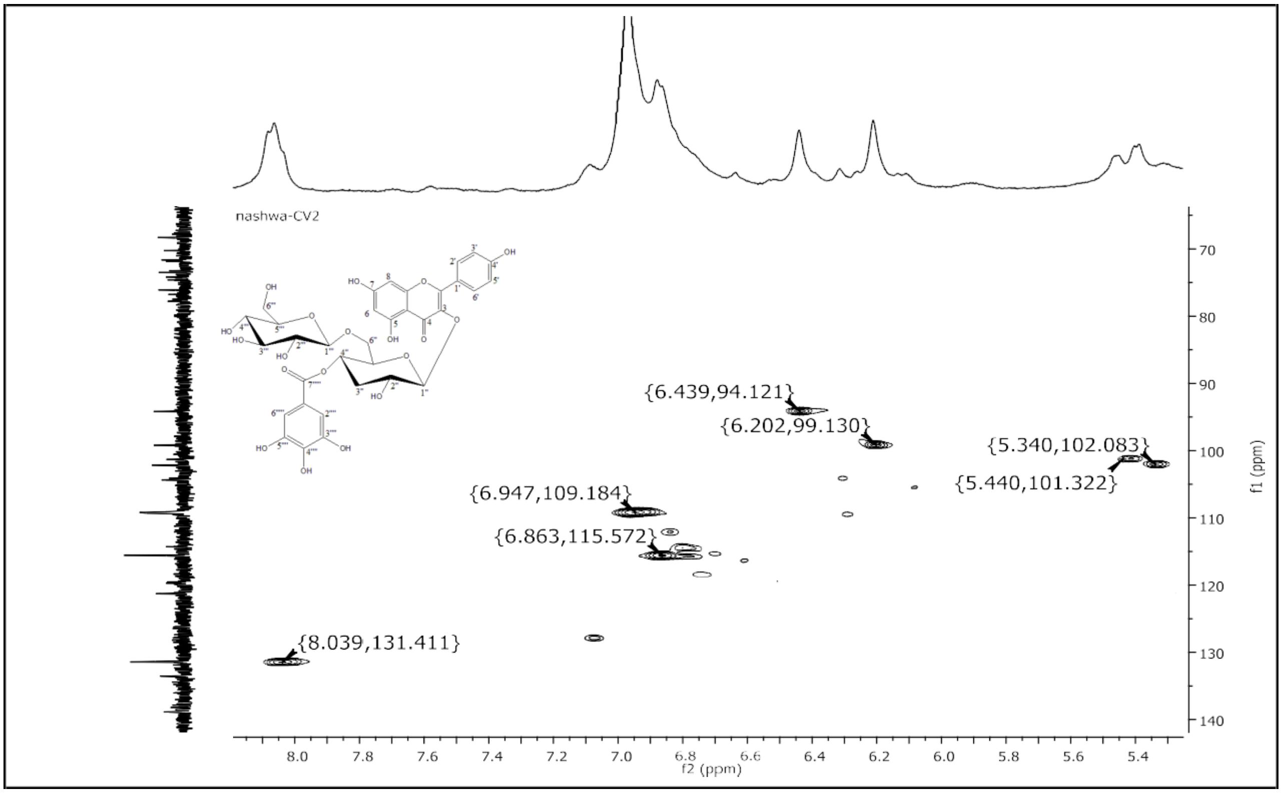

Fig. S3a: HSQC spectrum of compound **1** (400 MHz, DMSO-d_6)

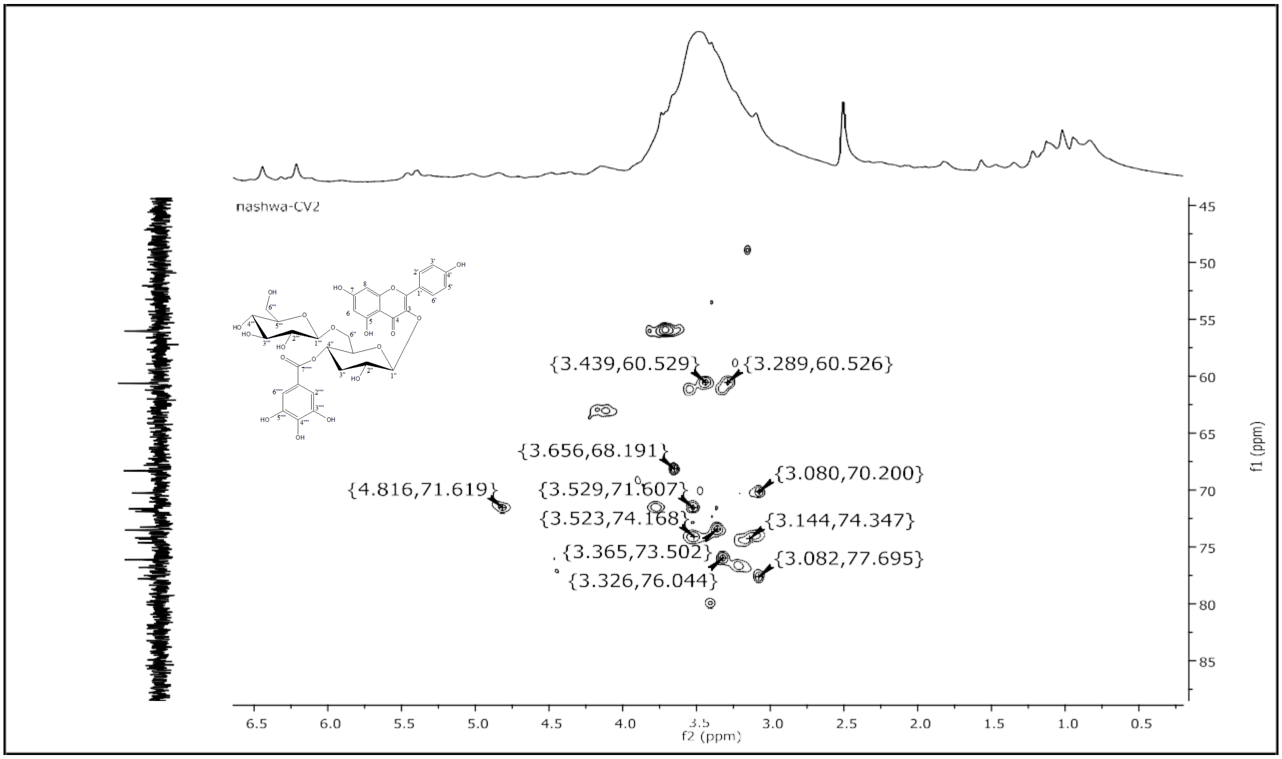

Fig. S3b: HSQC spectrum of compound **1** (400 MHz, DMSO-d_6)

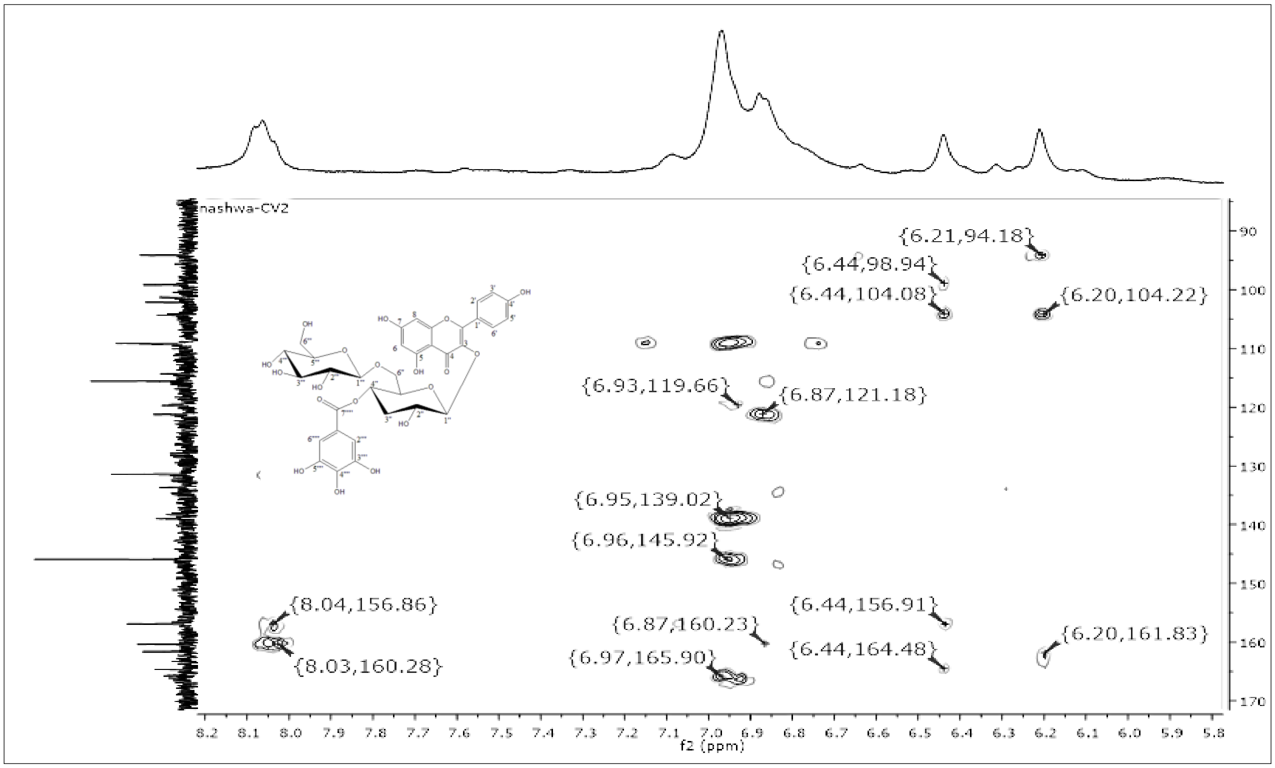

Figure S4a: HMBC spectrum of Compound **1** (400 MHz, DMSO-d_6)

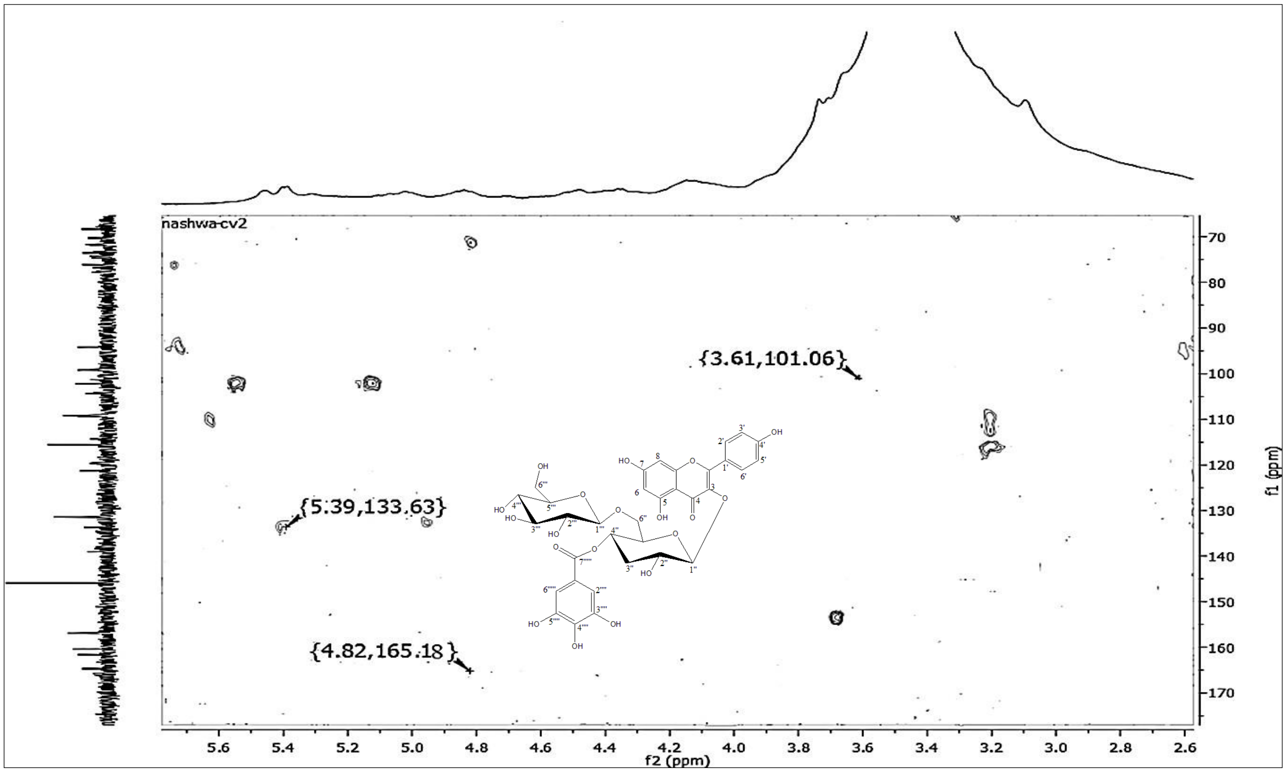

Figure S4b: HMBC spectrum of Compound **1** (400 MHz, DMSO-d_6)

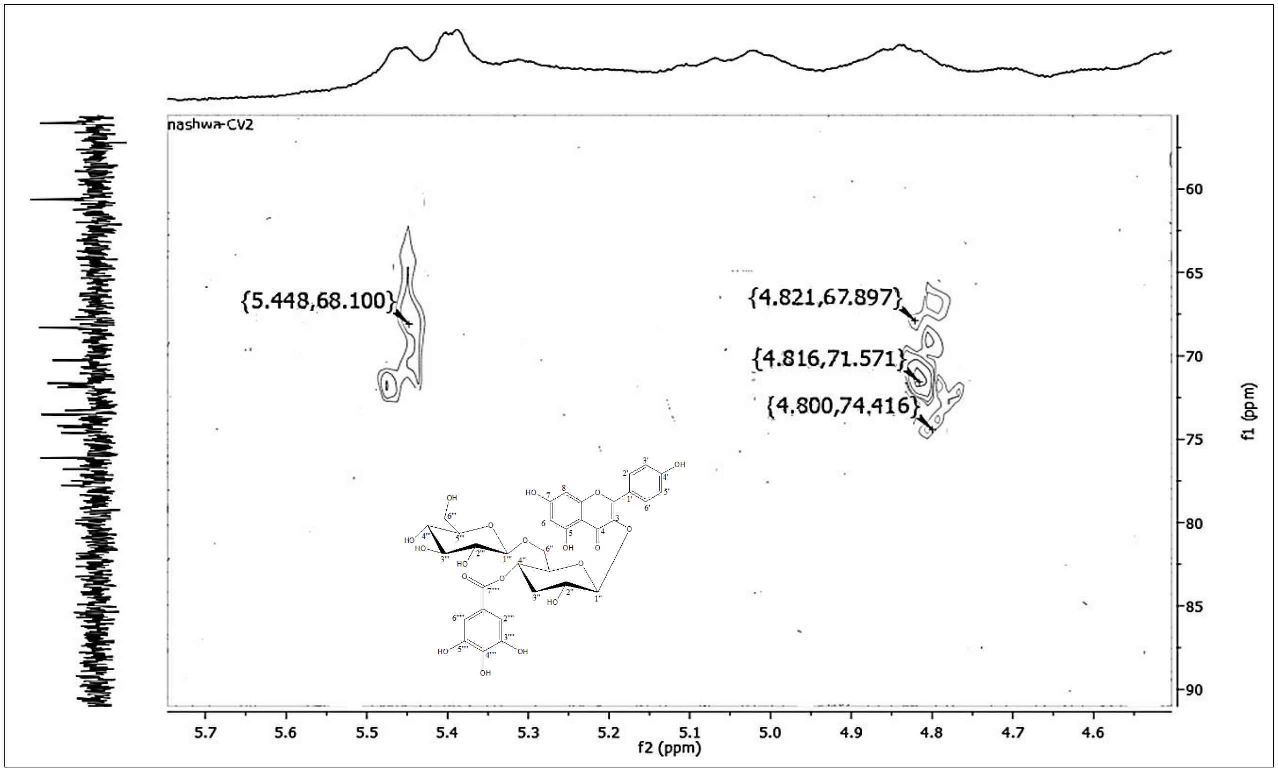

Figure S4c: HMBC spectrum of Compound **1** (400 MHz, DMSO-d_6)

.

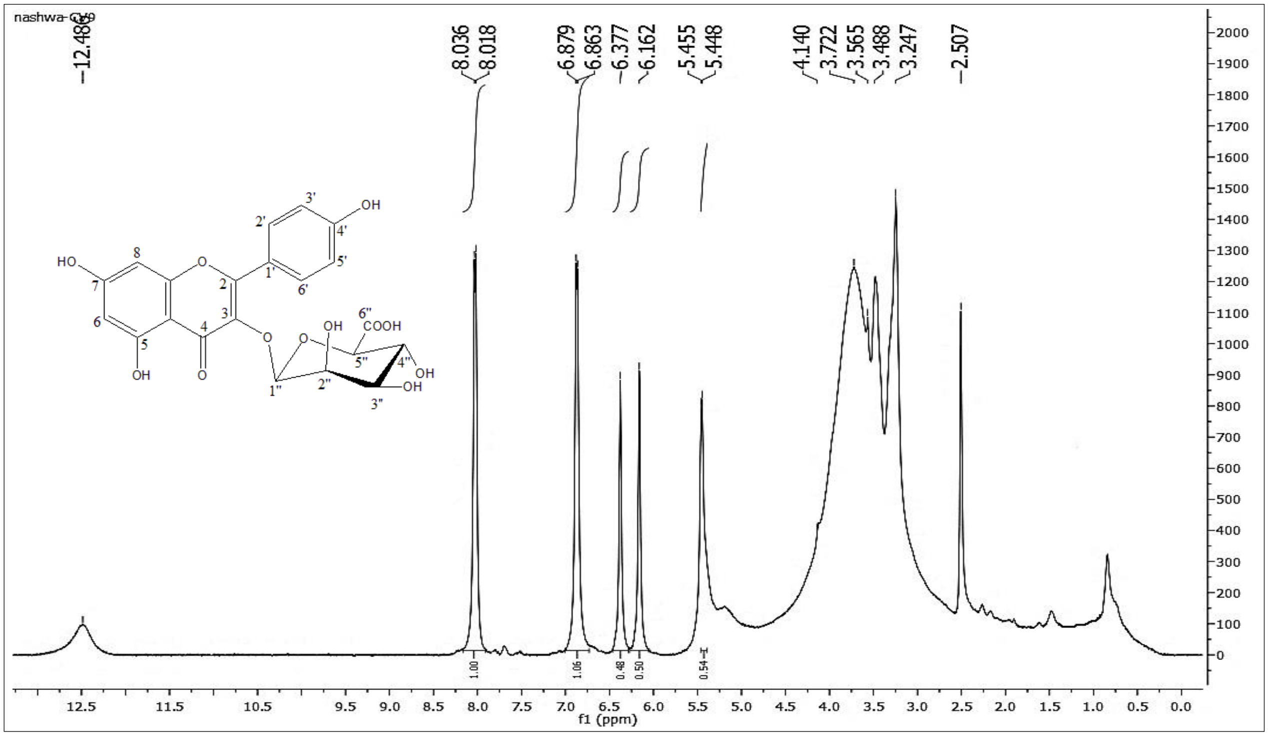

Figure S5: ^{1}HNMR spectrum of compound **2** (400 MHz, DMSO-d_6)

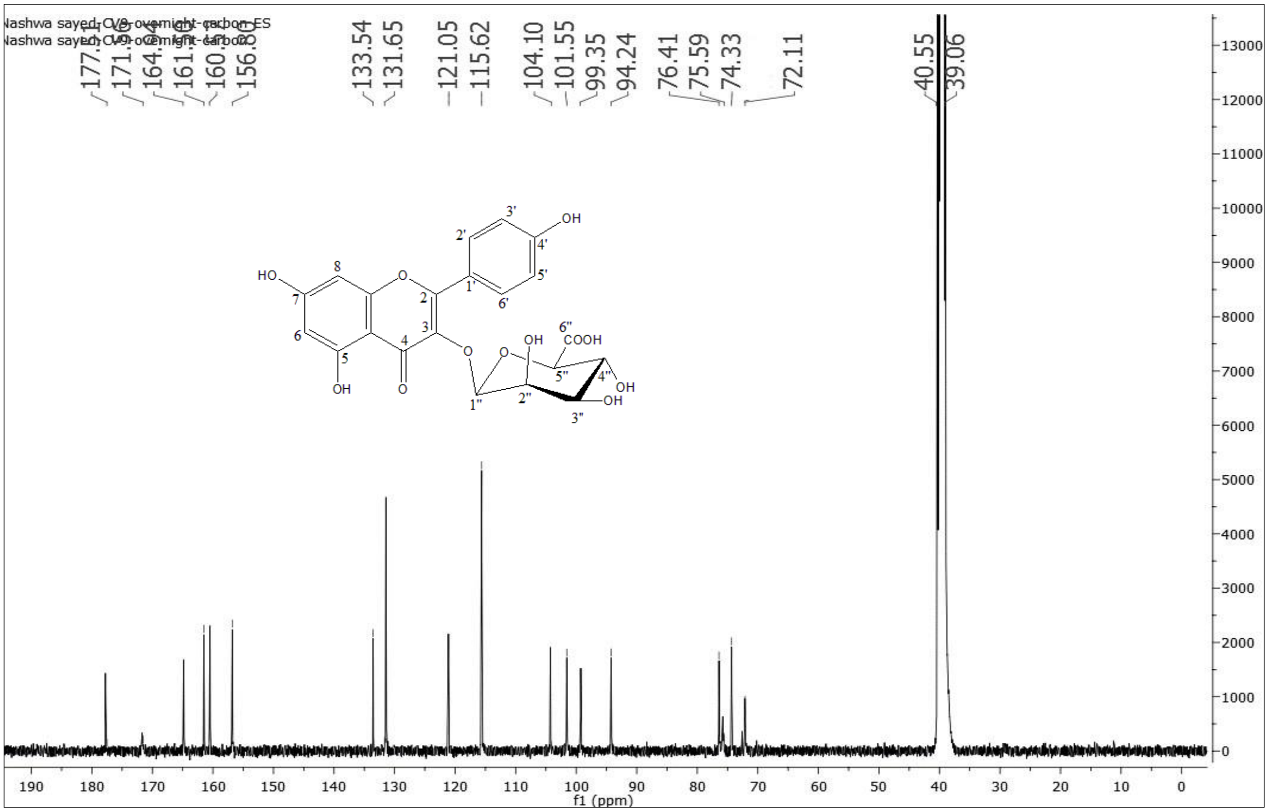

Figure S6: ^{13}CNMR spectrum of compound **2** (400 MHz, DMSO-d_6)

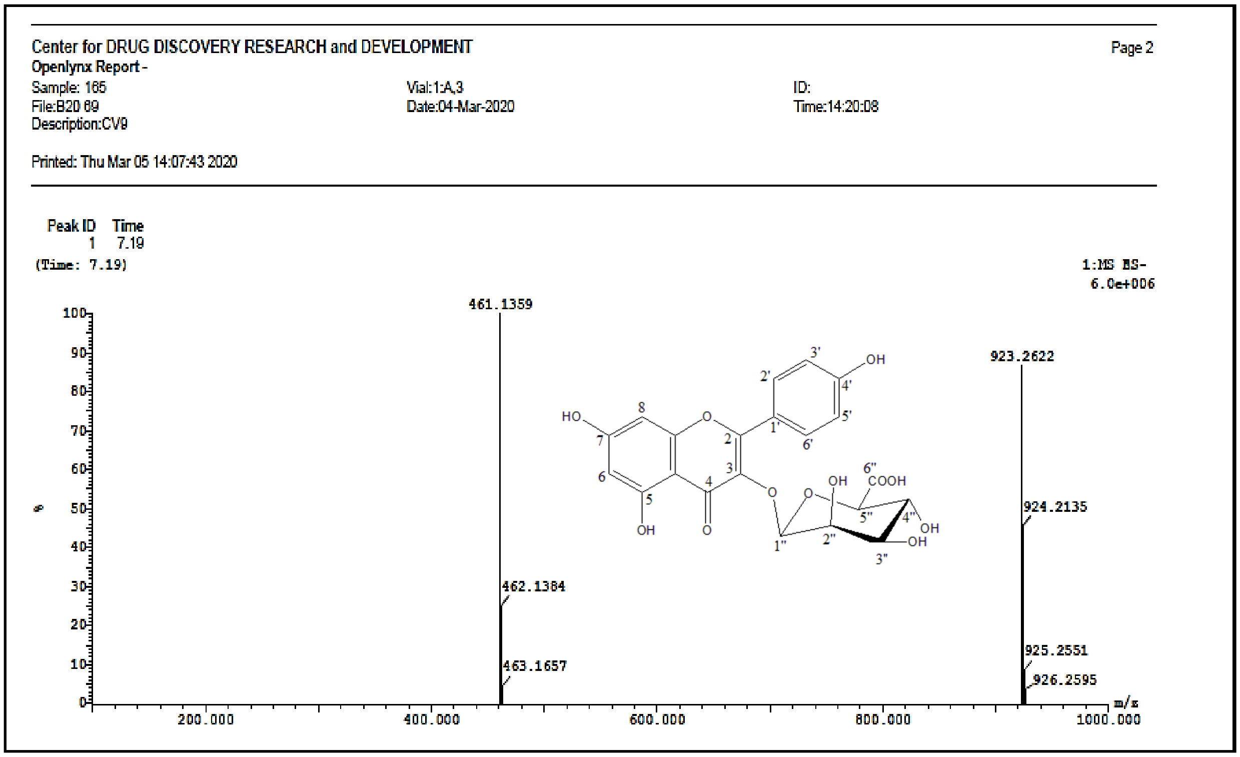

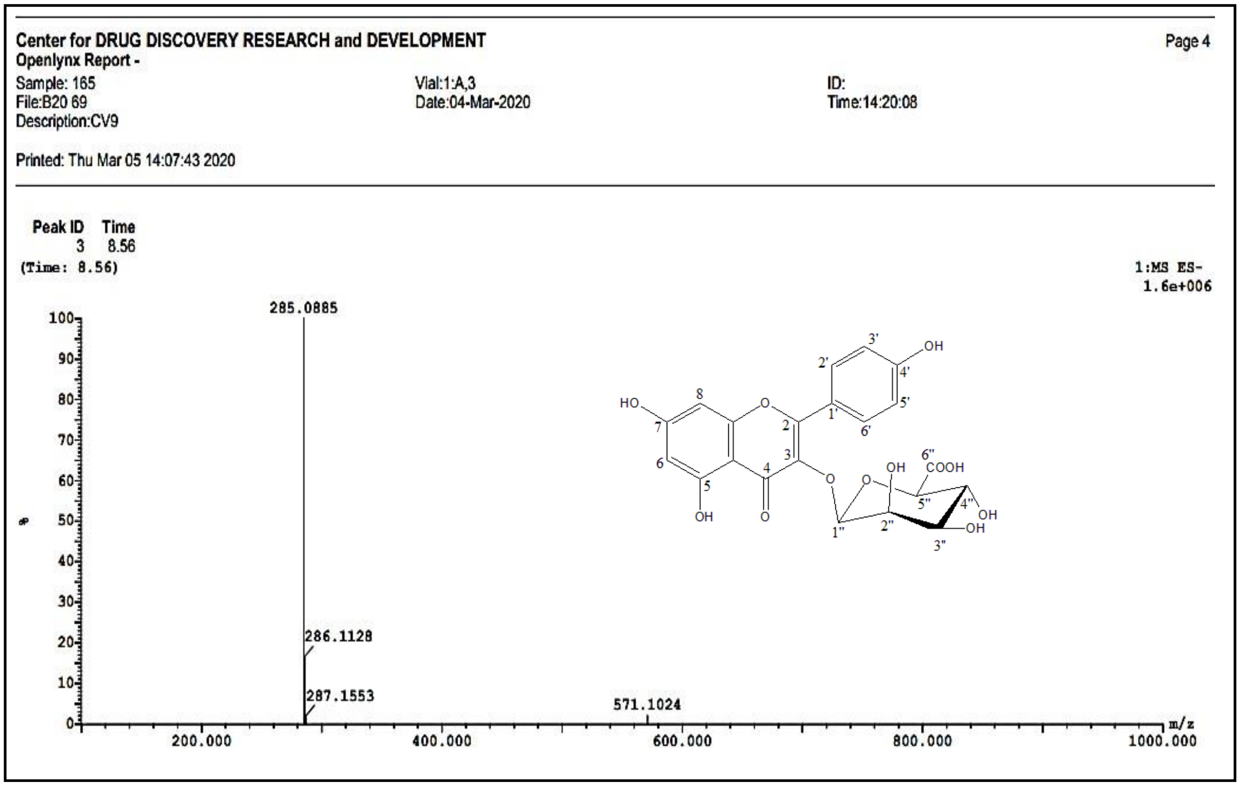

Fig. S7: Negative ESI/MS spectrum of compound **2**

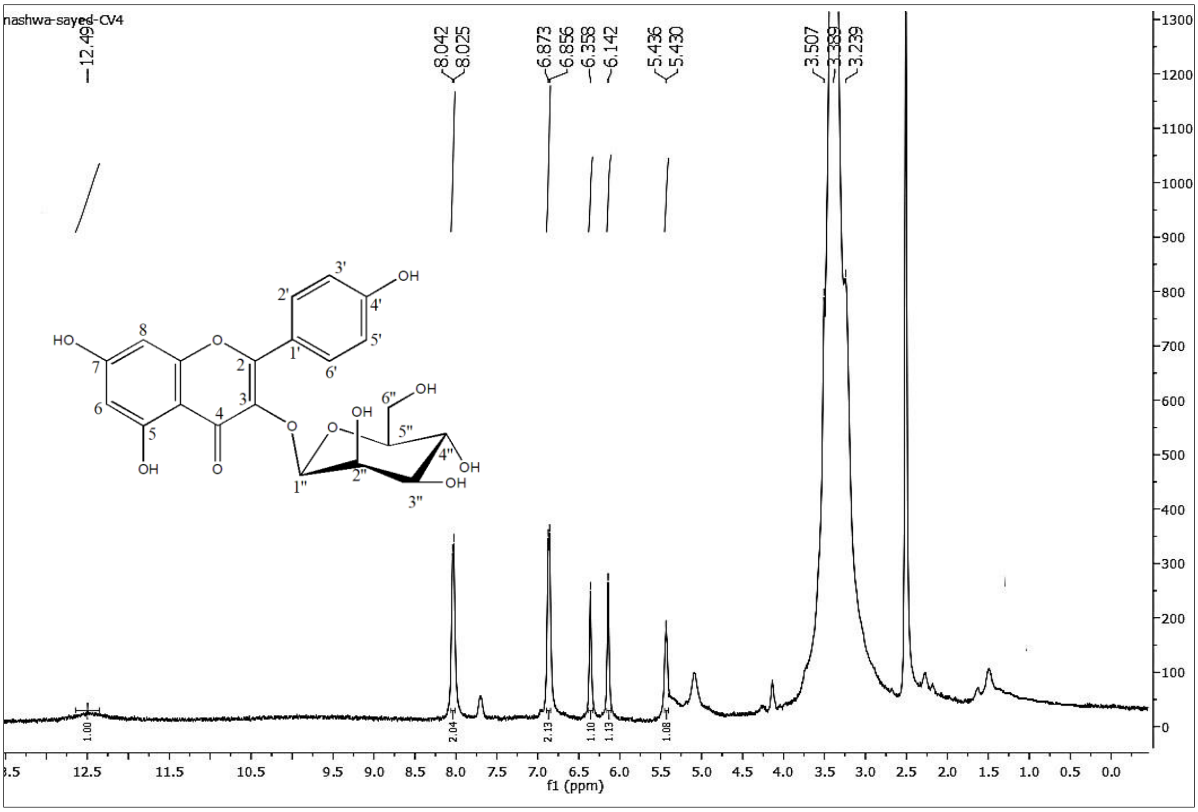

Figure S8: ^{1}HNMR spectrum of Compound **3** (400 MHz, DMSO-d_6)

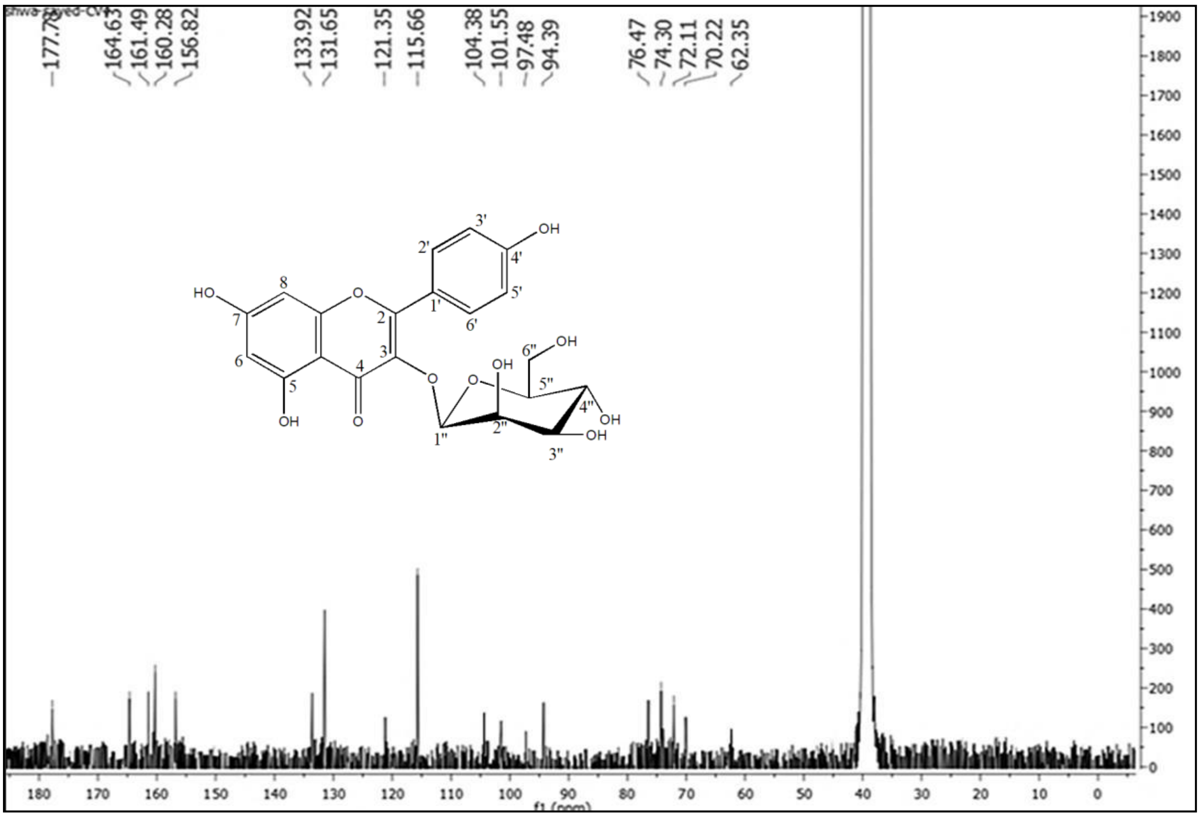

Figure S9: ^{13}CNMR spectrum of Compound **3** (400 MHz, DMSO-d_6)

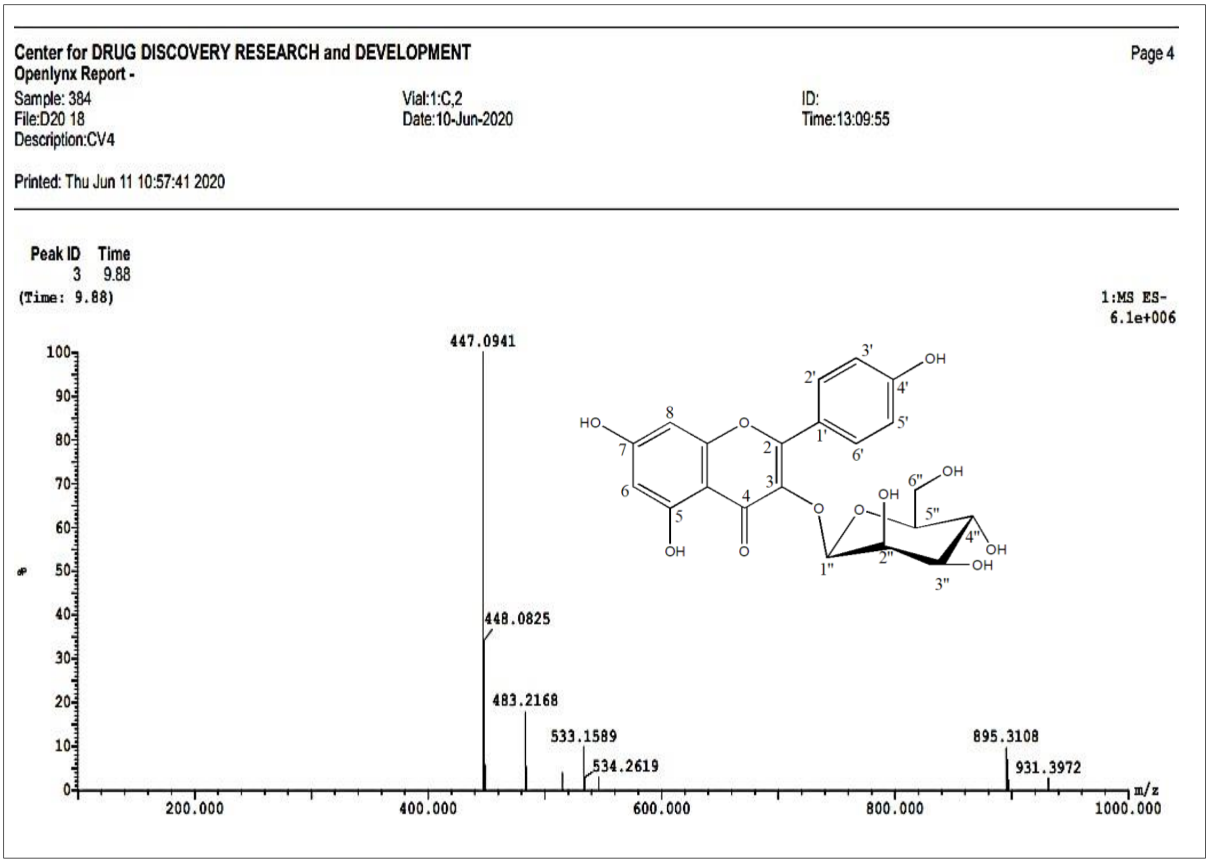

Figure S10: negative ESI / MS of compound **3**

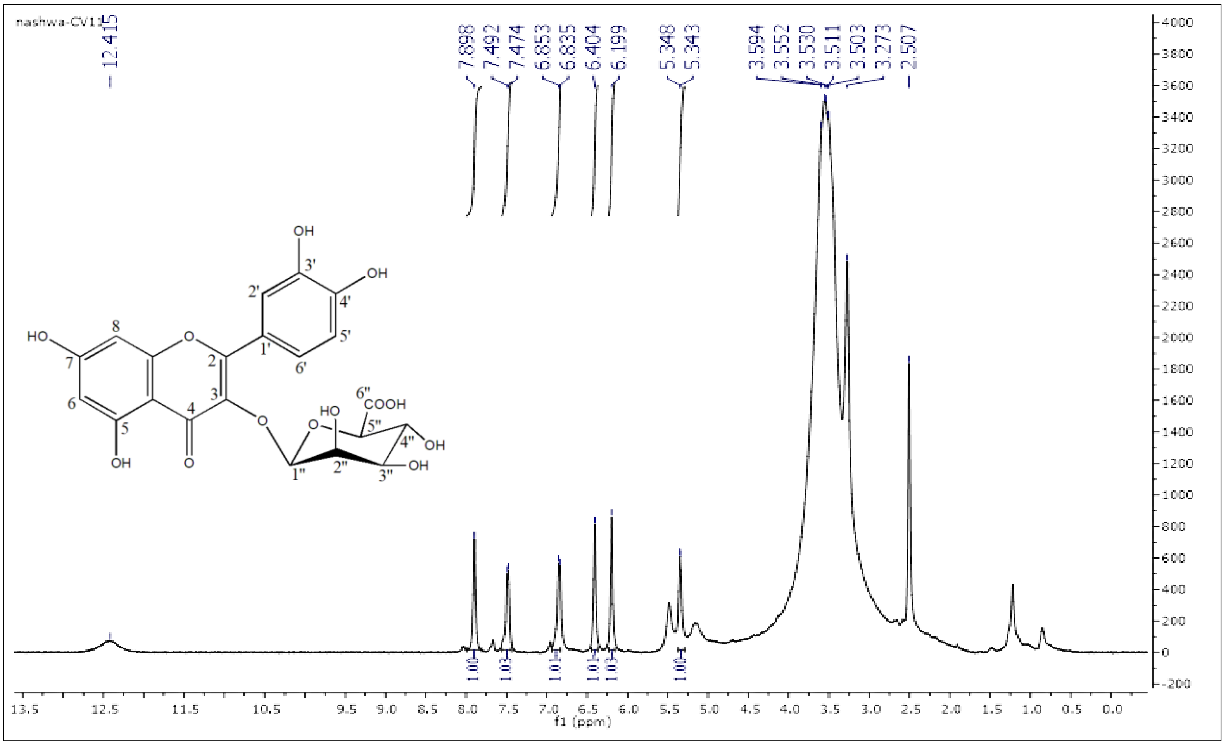

Figure S11: ^{1}HNMR spectrum of compound **4** (400 MHz, DMSO-d_6)

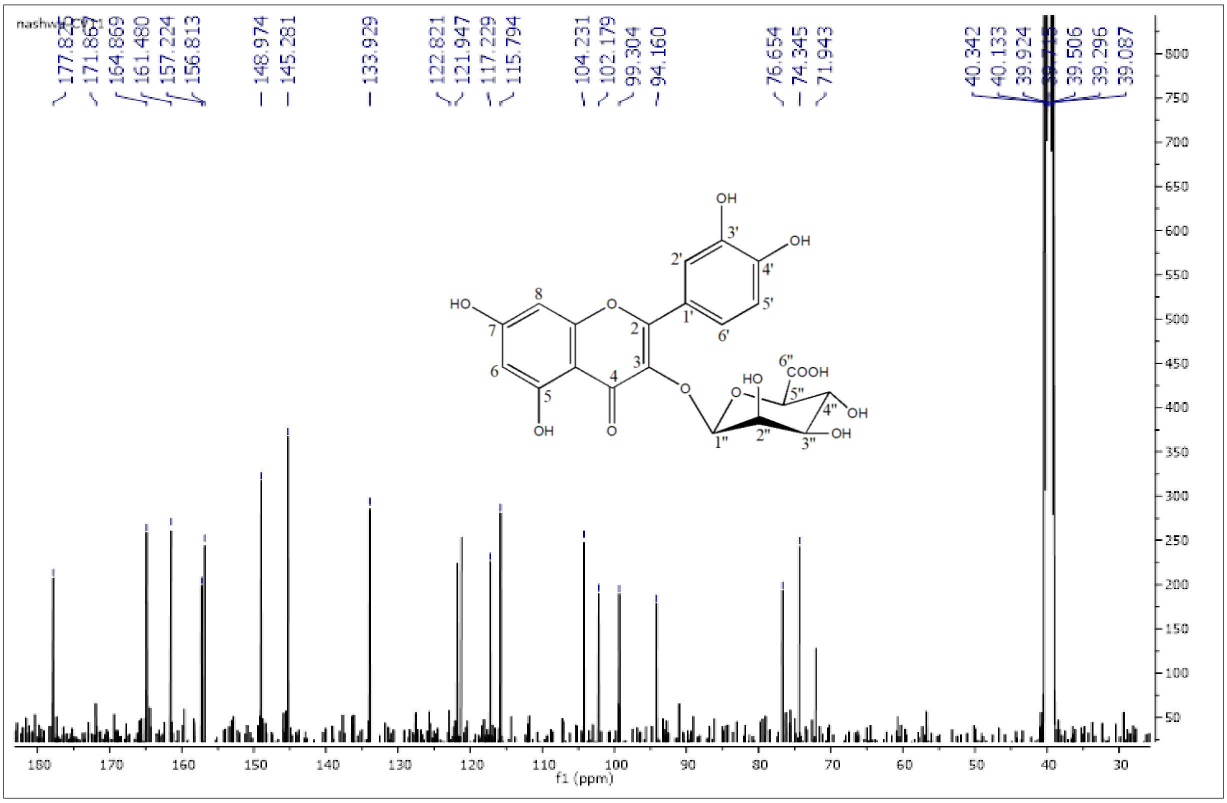

Figure S12: ^{13}CNMR spectrum of compound **4** (400 MHz, DMSO-d_6)

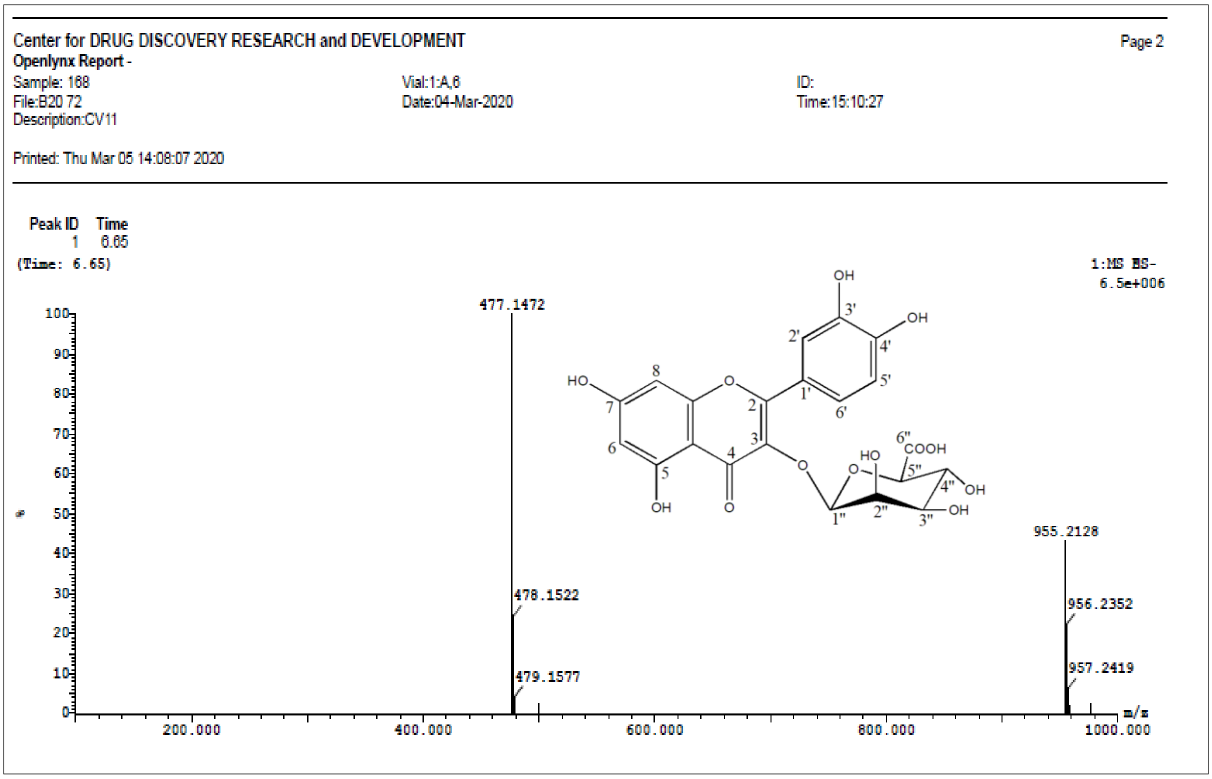

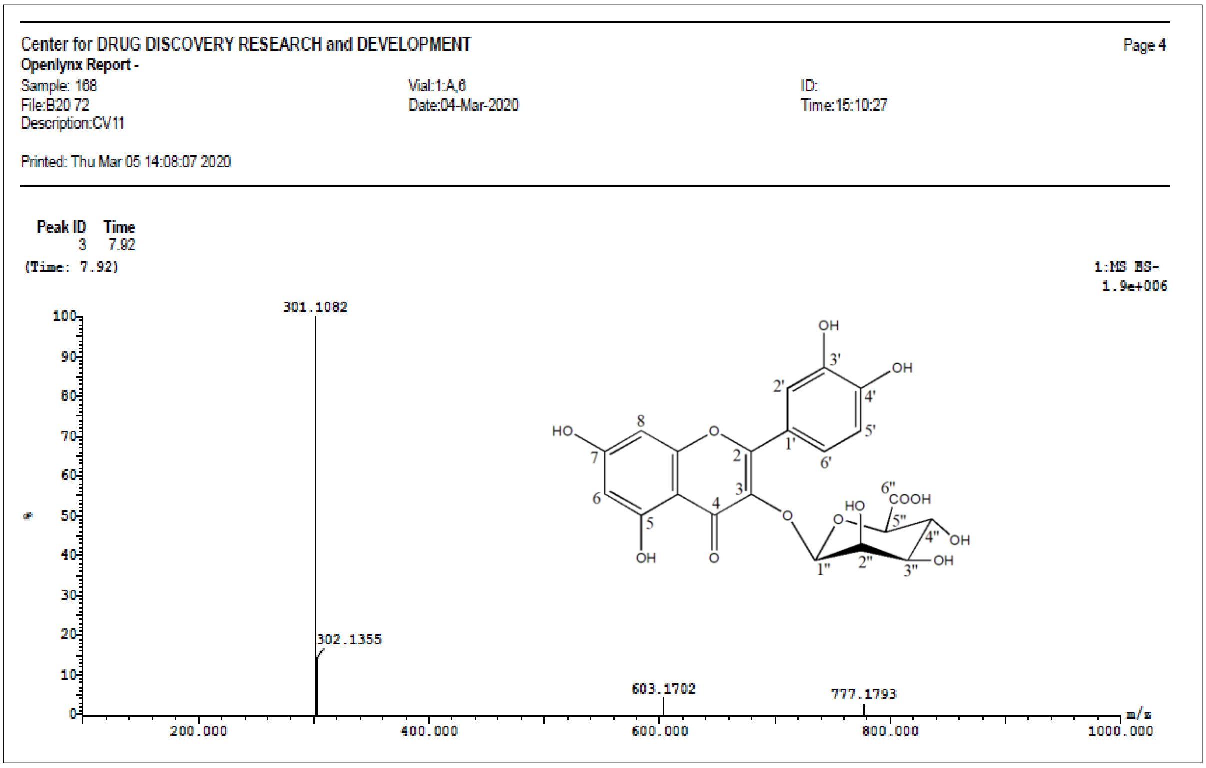

Figure S13: negative ESI / MS of compound **4**

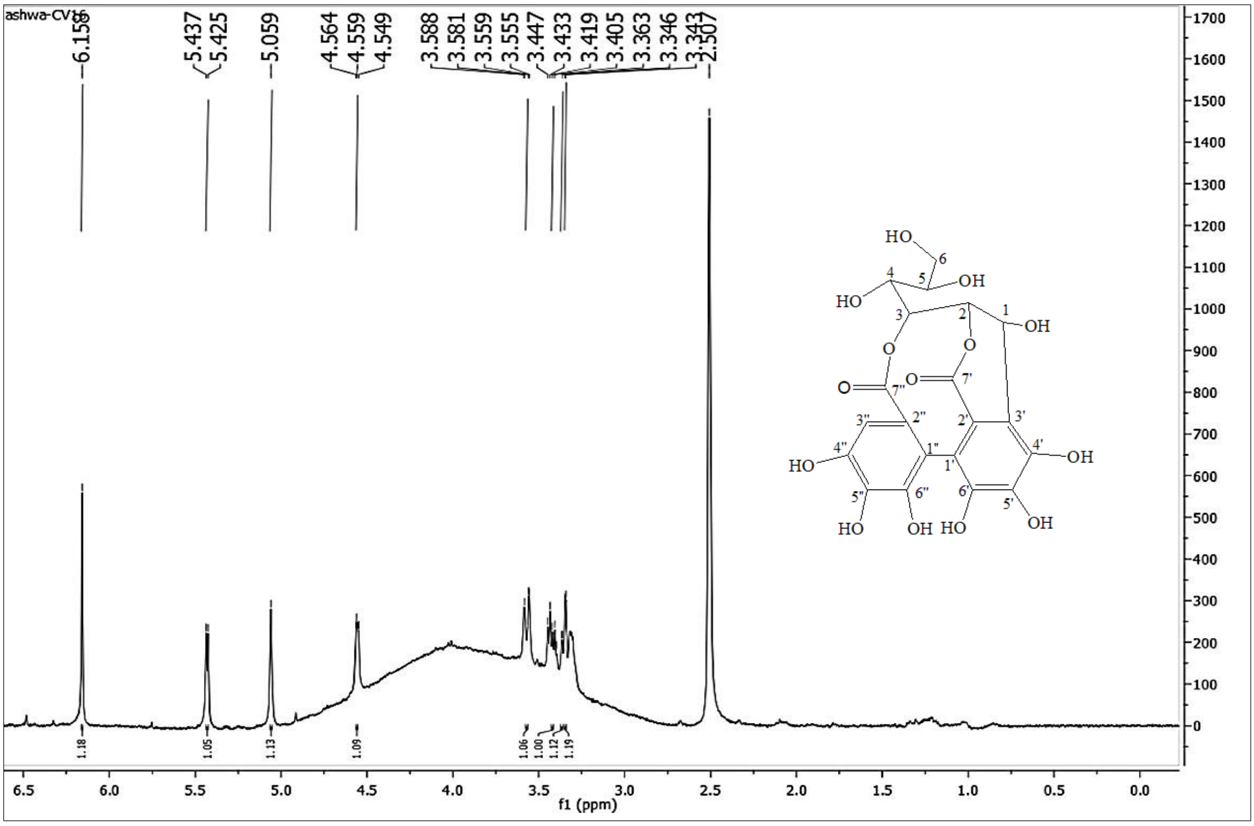

Figure S14: ^{1}HNMR spectrum of compound **5** (400 MHz, DMSO-d_6)

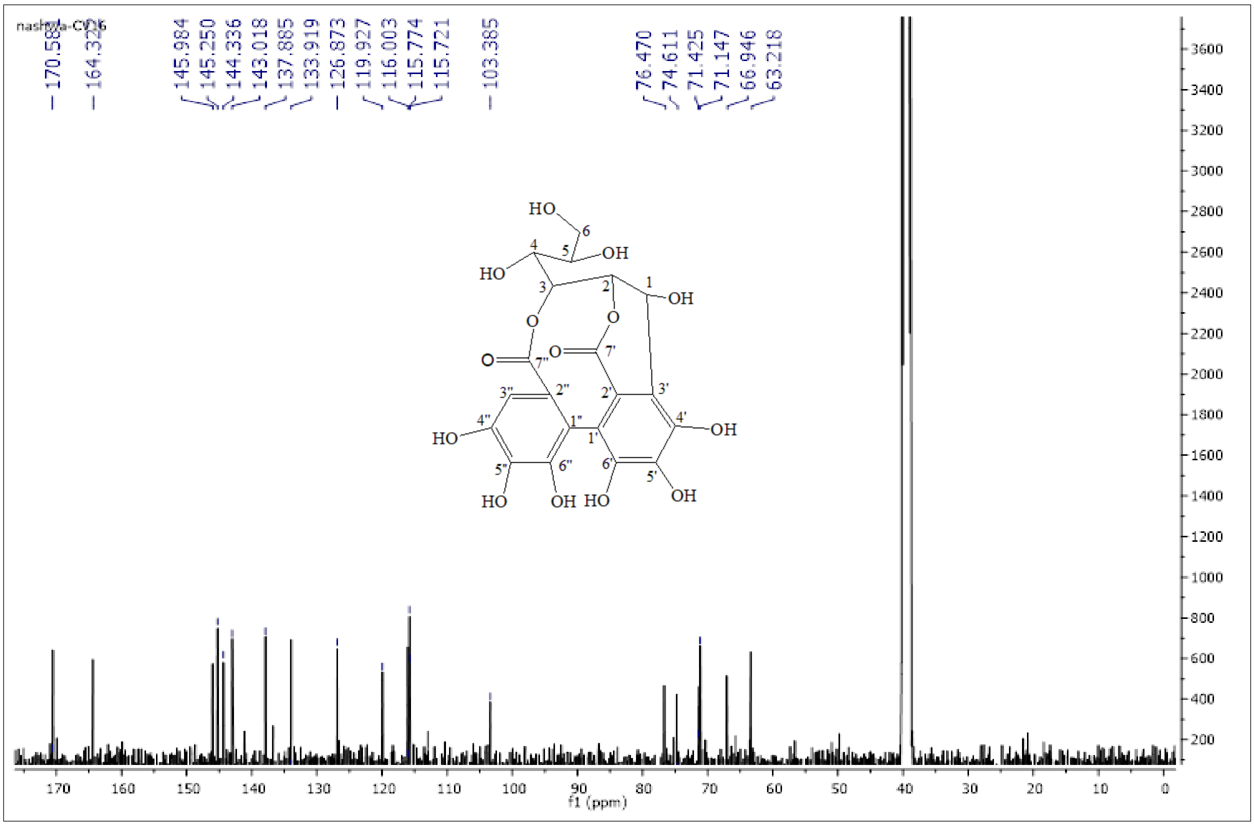

Figure S15: ^{13}CNMR spectrum of compound **5** (400 MHz, DMSO-d_6)

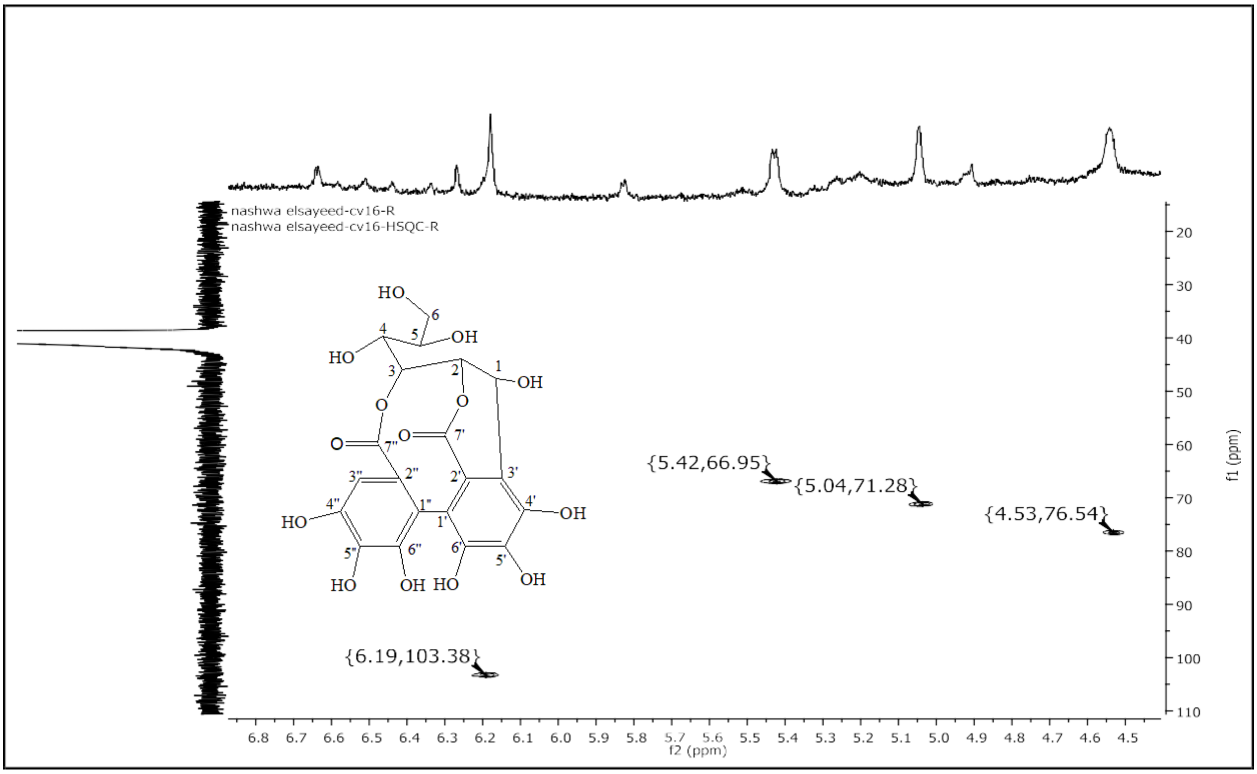

Fig. S16a: HSQC spectrum of compound **5** (400 MHz, DMSO-d_6)

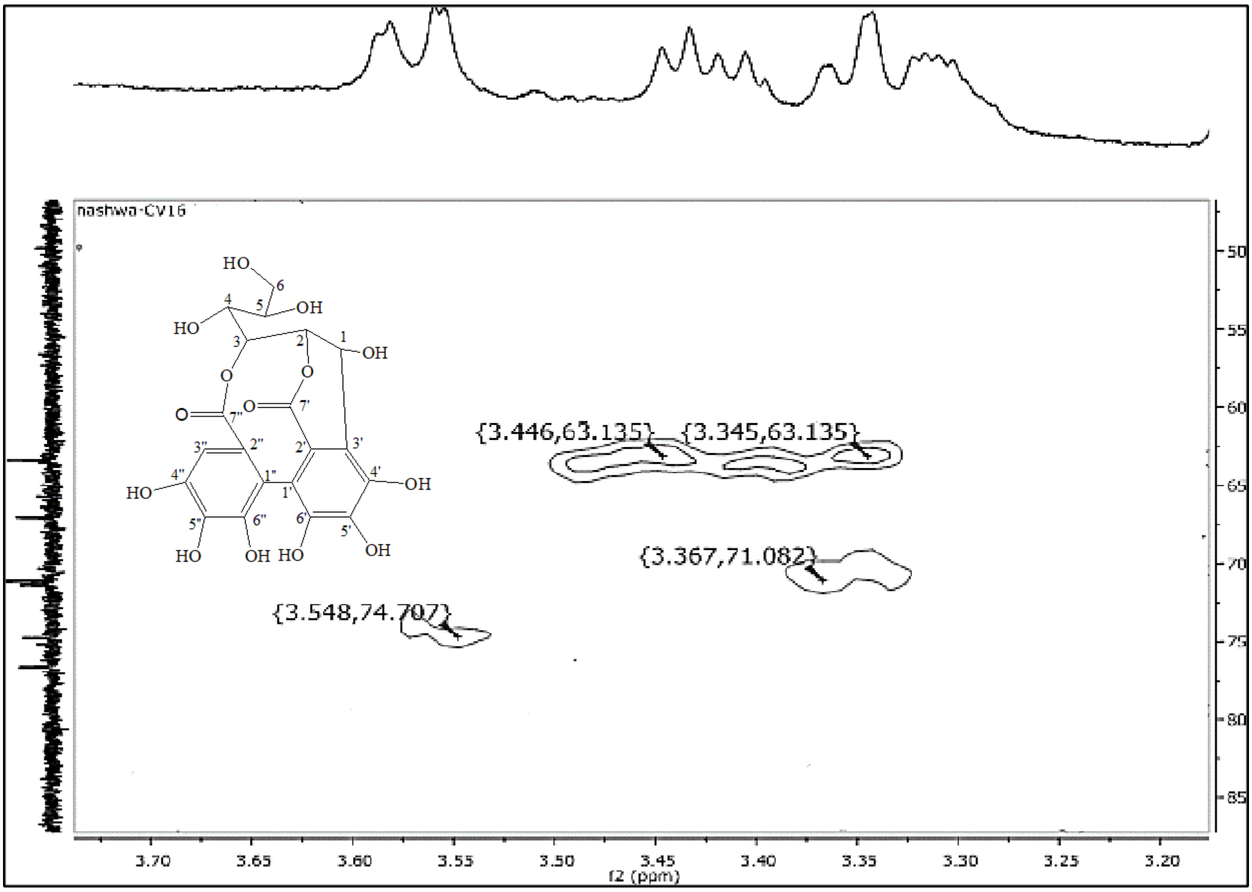

Fig. S16b: HSQC spectrum of compound **5** (400 MHz, DMSO-d_6)

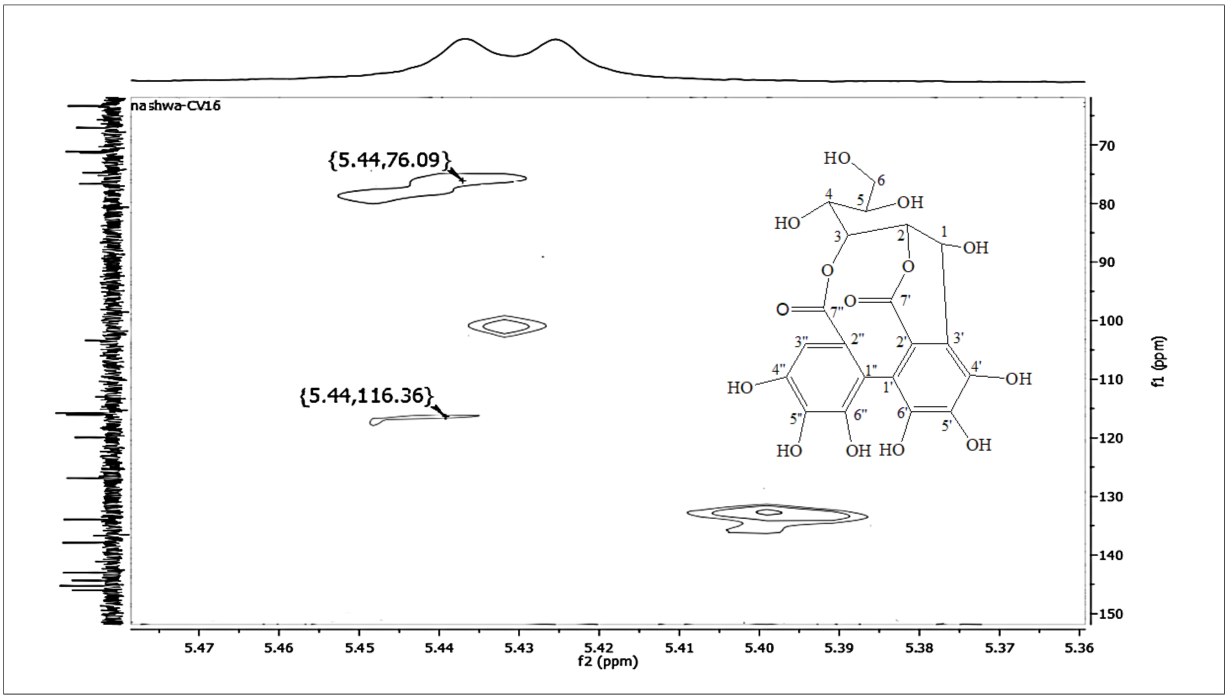

Figure S17a: HMBC spectrum of Compound **5**

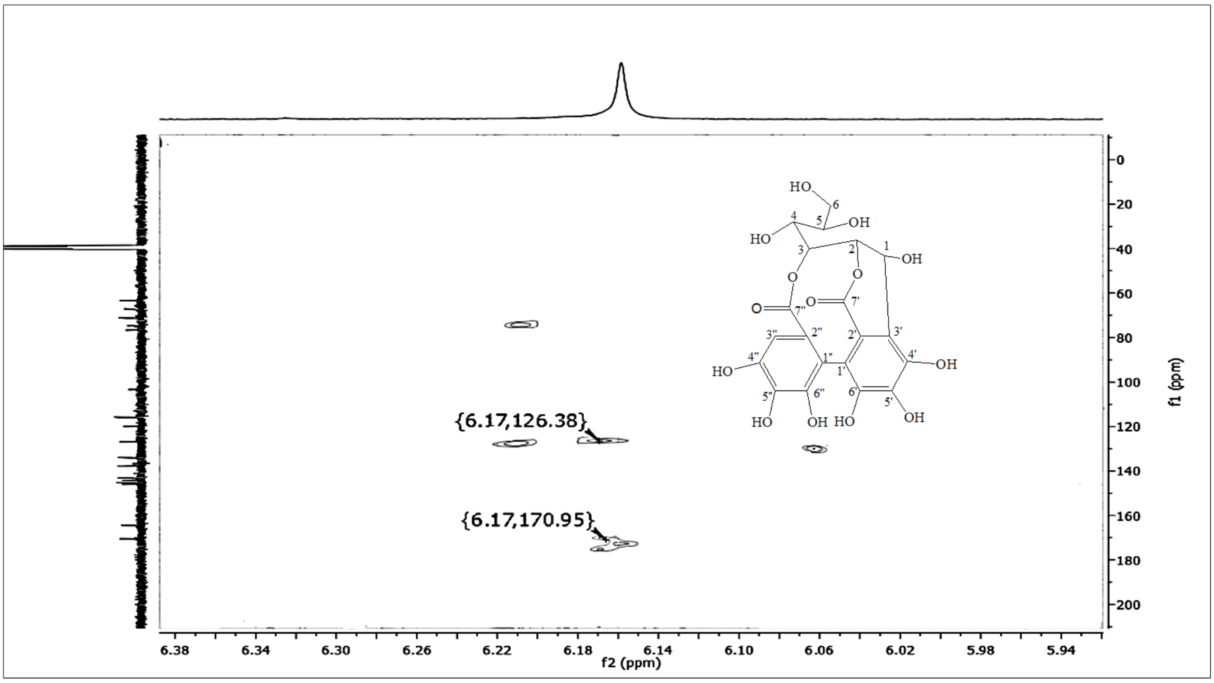

Figure S17b: HMBC spectrum of Compound **5**

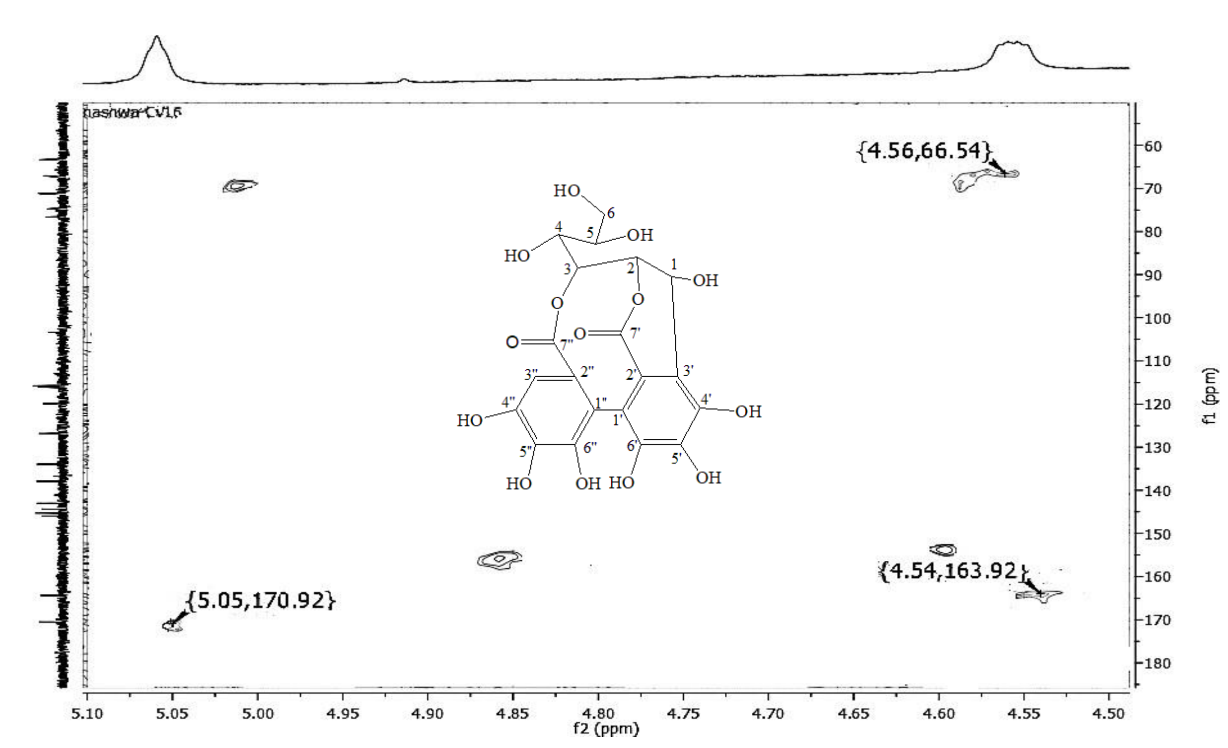

Figure S17c: HMBC spectrum of Compound **5**

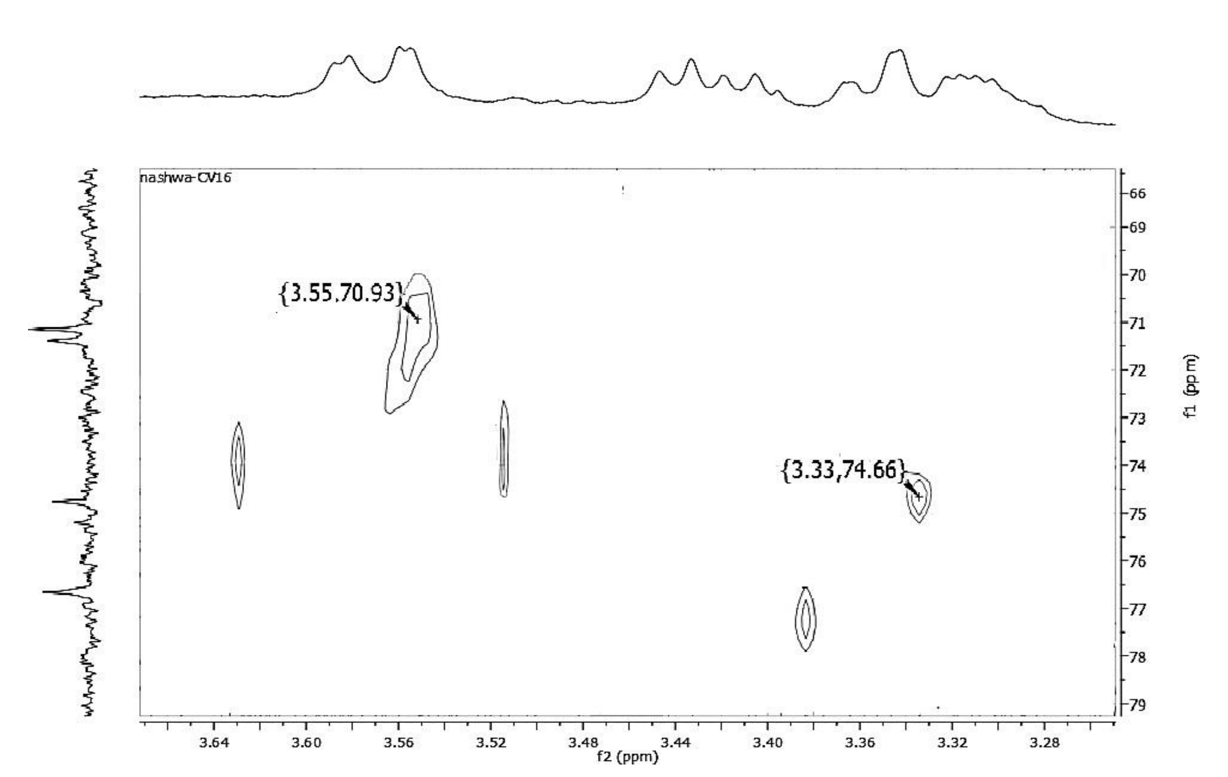

Figure S17d: HMBC spectrum of Compound **5**

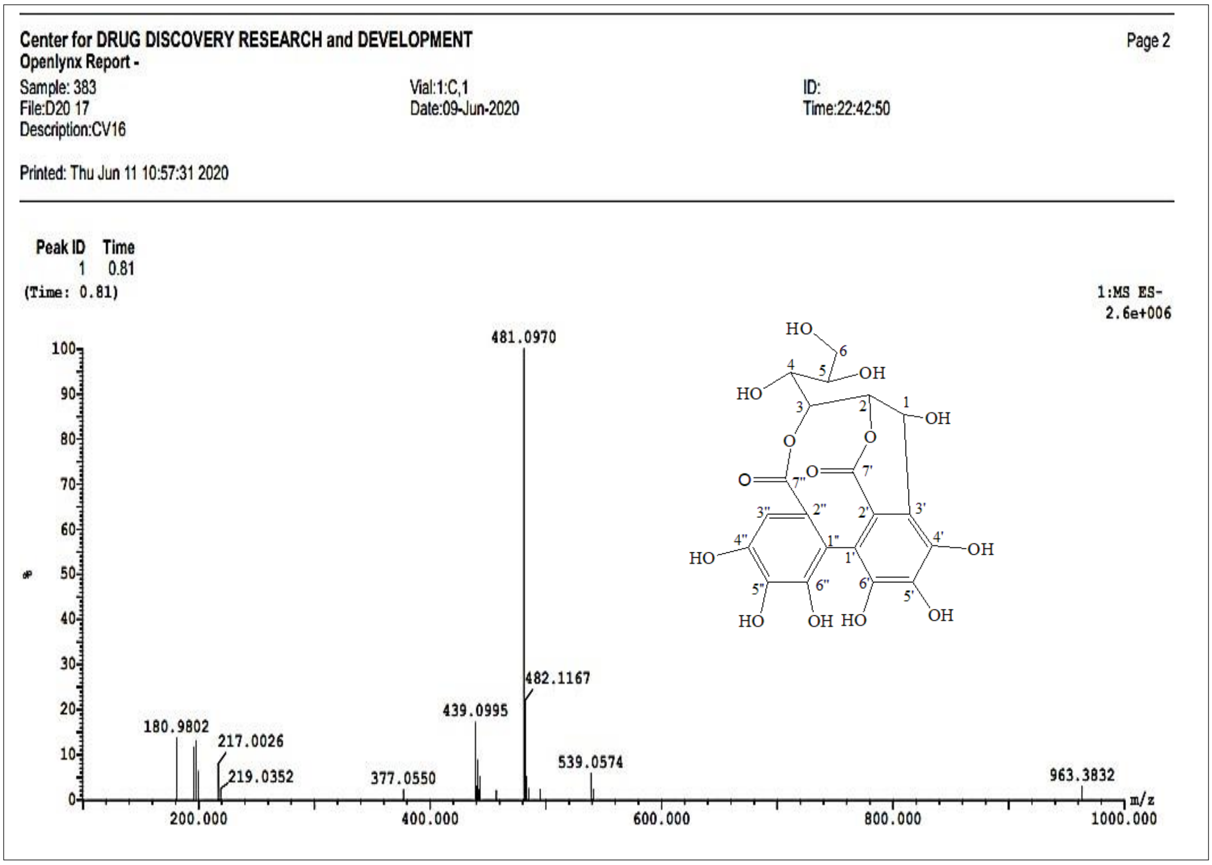

Figure S18: negative ESI / MS of compound **5**

B. Docking supplementary:

NDGA was found to form several non-covalent interactions with several close amino acid residue such as forming H-bonds with His600 (3.3 Å) and Arg596 (2.8 Å). In addition, some van der Waals with His372, Phe359 and Trp599. The docking results showed 9 possible conformers to NDGA with affinity ranging from -6.5 to -7.1 kcal/mol. Additionally, Compound **3** was docked inside the 5-LOX and 9 conformers were obtained with moderate affinity ranging from -6.4 to -7.4 kcal/mol. Compound **3** was found to form fewer non-covalent interactions inside the 5-LOX active site (Figure 4b): 1) H-bonds with Thr364 (2.77 Å), Pro569 (2.81 Å) and Arg596 (2.68 Å); 2) Van der Waals interactions with Phe359, His367, His372, Trp599, Ala603 and Leu607.

a)

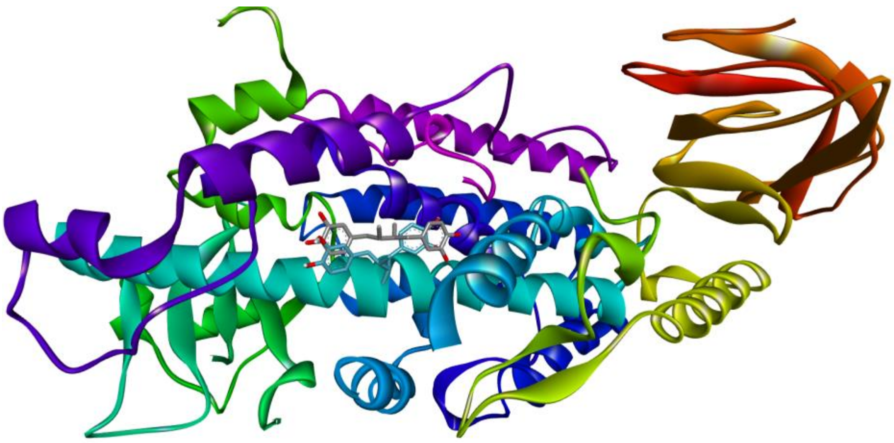

b)

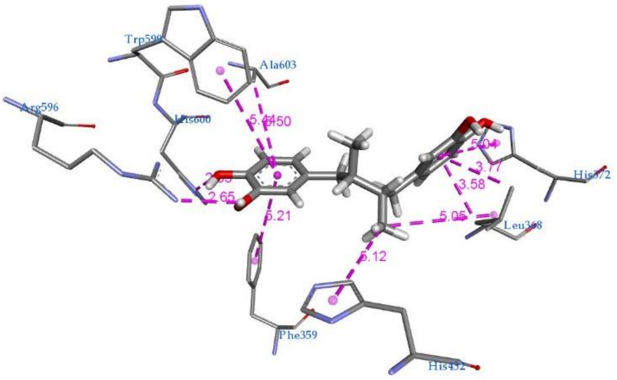

c)

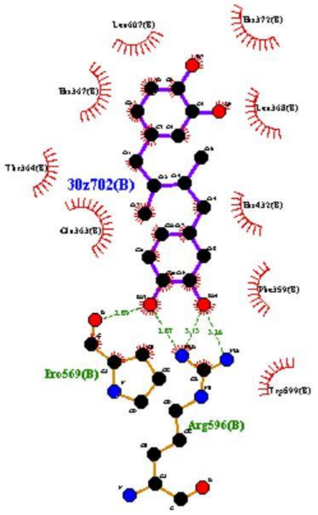

Figure S19a. a) Overlaying the crystal structure of NDGA (grey) and its modeled structure (greenish blue) inside the 5-LOX active site; b) 3D modeled structure of NDGA inside the 5-LOX active site; c) 2-D schematic representation for its non-covalent interactions inside the 5-LOX active site.

a)

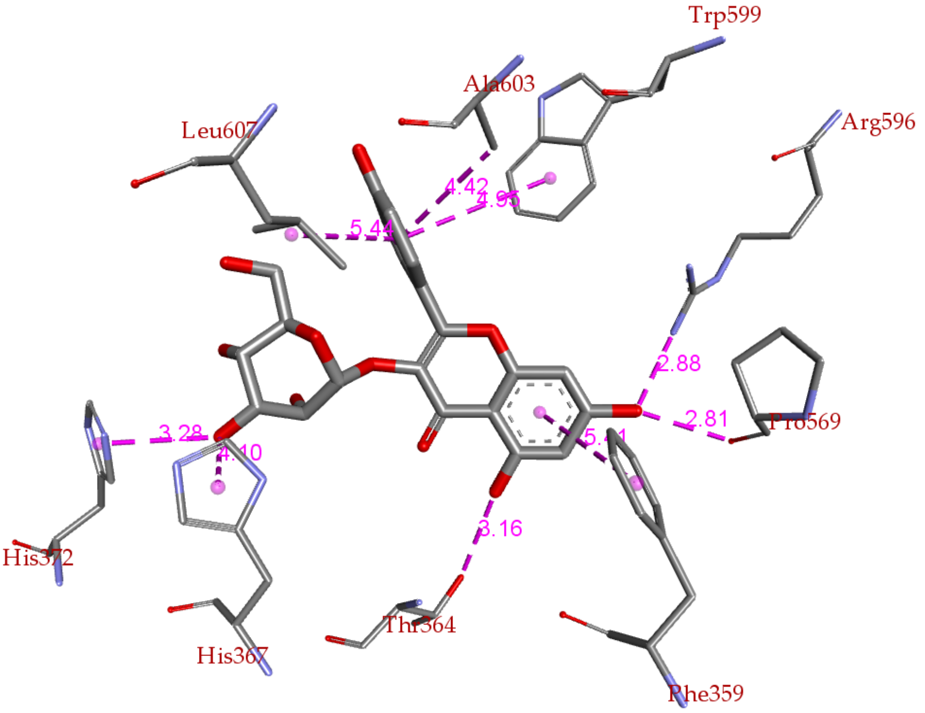

b)

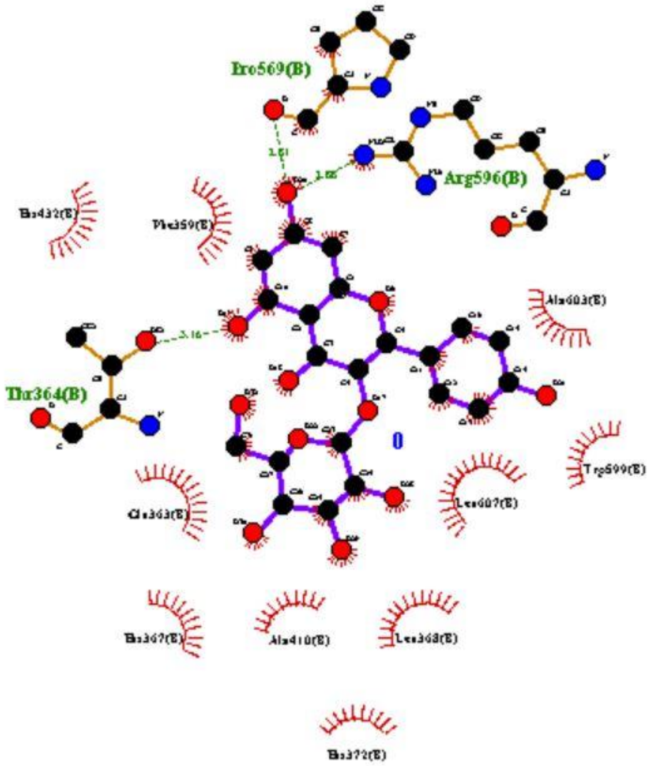

Figure S19b. a) Docking of compound **3** inside the 5-LOX active site and 2-D schematic representation for its non-covalent interactions inside 5-LOX active site; b) Docking of compound **3** inside the 5-LOX active site and 2-D schematic representation for its non-covalent interactions inside the 5-LOX active site.

mode	affinity (kcal/mol)	dist from best mode rmsd l.b.	rmsd u.b.
1	-7.1	0.000	0.000
2	-7.1	0.116	8.074
3	-6.9	1.514	2.300
4	-6.9	1.318	7.741
5	-6.8	3.161	5.028
6	-6.7	2.185	2.891
7	-6.6	1.359	8.131
8	-6.6	1.969	2.947
9	-6.5	1.883	3.070

Figure S20a. Docking results for NDGA inside 5-LOX active site.

mode	affinity (kcal/mol)	dist from best mode rmsd l.b.	rmsd u.b.
1	-8.0	0.000	0.000
2	-8.0	2.505	7.960
3	-7.8	2.575	3.657
4	-7.7	2.002	6.784
5	-7.7	2.530	5.136
6	-7.5	1.754	6.811
7	-7.4	3.490	7.671
8	-7.2	2.826	4.931
9	-7.1	2.041	6.680

Figure S20b. Docking results for compound **2** inside 5-LOX active site.

mode	affinity (kcal/mol)	dist from best mode rmsd l.b.	rmsd u.b.
1	-7.4	0.000	0.000
2	-7.3	2.319	4.913
3	-7.3	2.034	6.695
4	-6.7	2.352	7.304
5	-6.6	1.818	3.284
6	-6.6	2.821	7.271
7	-6.4	2.006	4.026
8	-6.4	2.543	4.517
9	-6.4	2.015	6.657

Figure S20c. Docking results for compound **3** inside 5-LOX active site.

mode	affinity (kcal/mol)	dist from best mode rmsd l.b.	rmsd u.b.
1	-8.1	0.000	0.000
2	-7.5	1.877	2.872
3	-7.4	2.825	7.653
4	-7.3	2.433	7.333
5	-7.2	2.484	8.451
6	-7.2	1.785	6.725
7	-7.0	2.431	6.464
8	-7.0	2.225	6.674
9	-6.9	2.872	4.863

Figure S20d. Docking results for compound **7** inside 5-LOX active site.

mode	affinity (kcal/mol)	dist from best mode rmsd l.b.	rmsd u.b.
1	-8.3	0.000	0.000
2	-7.8	2.074	6.841
3	-7.7	2.653	3.977
4	-7.7	2.187	6.827
5	-7.7	2.316	6.678
6	-7.6	1.672	5.058
7	-7.5	2.746	7.079
8	-7.4	2.088	2.591
9	-7.2	1.571	2.349

Figure S20e. Docking results for compound **16** inside 5-LOX active site.

mode	affinity (kcal/mol)	dist from best mode rmsd l.b.	rmsd u.b.
1	-8.1	0.000	0.000
2	-7.4	2.329	8.667
3	-7.2	1.745	2.286
4	-7.2	2.501	9.705
5	-7.1	3.281	5.146
6	-7.0	1.155	2.269
7	-6.9	2.847	8.682
8	-6.9	1.722	9.380
9	-6.9	1.386	2.325

Figure S20f. Docking results for compound **17** inside 5-LOX active site.

mode	affinity (kcal/mol)	dist from best mode rmsd l.b.	rmsd u.b.
1	-4.9	0.000	0.000
2	-4.9	1.766	3.059
3	-4.8	8.900	10.524
4	-4.8	1.498	2.737
5	-4.8	1.568	3.659
6	-4.7	6.832	8.774
7	-4.7	1.178	2.070
8	-4.7	1.116	4.315
9	-4.6	6.182	7.542

Figure S20g. Docking results for ascorbic acid inside 5-LOX active site.

mode	affinity (kcal/mol)	dist from best mode rmsd l.b.	rmsd u.b.
1	-7.2	0.000	0.000
2	-7.1	0.782	1.540
3	-6.7	1.745	2.289
4	-6.7	2.434	3.003
5	-6.5	1.724	3.145
6	-6.2	17.353	21.547
7	-6.1	2.235	7.055
8	-6.1	2.272	3.742
9	-6.0	2.883	7.567

Figure S20h. Docking results for quercetin inside 5-LOX active site.
